# Composition and function of the nuchal hump of male *Xiphophorus multilineatus*

**DOI:** 10.1007/s10695-025-01539-2

**Published:** 2025-07-31

**Authors:** Keith Tompkins, Will Boswell, Kang Du, Zhao Lai, Yuan Lu, Molly R. Morris

**Affiliations:** 1 Department of Biological Sciences, Ohio University, Athens, OH 45701 USA; 2 Institute for Molecular Life Sciences, Texas State University, San Marcos, TX 78666 USA; 3 Department of Molecular Medicine, University of Texas Health San Antonio, San Antonio, TX 78229 USA; 4 Genome Sequencing Facility, Greehey Children’s Cancer Research Institute (GCCRI), University of Texas Health San Antonio, San Antonio, TX 78229 USA

**Keywords:** Nuchal hump, Lipid storage, Temperature, *Xiphophorus multilineatus*

## Abstract

Nuchal humps are morphological traits that occur among vertebrate species and have multiple functions. The emergence of nuchal humps in *Xiphophorus multilineatus* males maintained in the laboratory, a species that does not develop humps in the wild, provided a unique opportunity to explore the development and function of this trait. The current study investigated the tissue composition of the hump and analyzed the influence of rearing temperature and diet restriction on hump development. Through histological examination and gene expression profiling, our findings show that the hump is composed primarily of adipose tissue, which suggests a role in energy storage via fat deposition. Nuchal humps grew to a larger size in the cold environment (20 °C) than in the warm (25 °C), and the differential gene expression pattern between temperature treatments suggests increased fat deposition in the cold versus the warm environment. For example, the G0S2 gene, which inhibits lipid catabolism, is upregulated in the cold environment, and the WARS-1 gene, which leads to increased fat stores when under-expressed, is upregulated in the warm environment. The hypothesis that temperature influences hump development by stimulating shifts in fat metabolism is further supported by the finding that males from the warm environment used fat stores in the nuchal hump at a faster rate when placed on a restricted diet than males from the cold. The influence of temperature, diet, and potentially activity levels on fat deposition suggests *X. multilineatus* as an evolutionary animal model for gaining insights into the mechanisms involved in human obesity.

**Supplementary Information:**

The online version contains supplementary material available at 10.1007/s10695-025-01539-2.

## Introduction

Nuchal humps, broadly defined as a tissue mass located dorsal posterior to the head, are present across multiple vertebrate taxa. The tissue composition of nuchal humps varies among vertebrate species, as does the development and function of this structure (Alexander and Player 1965; Cave and Allbrook 1958; Manamendra-Arachchi et al. 2006; Susuki et al. 2014; Ward and Ward 2020). In sexually monomorphic fish species where the hump is persistent, such as *Gila cypha*, an anti-predatory function is likely. Both sexes would benefit from the presence of a nuchal hump due to increased body depth, which can limit the ability of predators to capture individuals once the hump develops (Douglas 1993; Portz and Tyus 2004); Ward and Ward 2020). Among sexually dimorphic species, nuchal humps are often sexually selected traits, and proposed functions include species recognition, sex recognition, and indication of mate quality (Barlow and Siri 1997; Takahashi 2018; Rometsch et al. 2021). There is also evidence to suggest that the nuchal hump may play a role in energy storage, based on increased expression of genes known to promote adipogenesis and cell proliferation within nuchal hump tissue of the African cichlid *Cyrtocara moorii* (Lecaudey et al. 2019), and enlargement of the hump in another African cichlid, *Cyphotilapia gibberosa*, commonly known as Mpimbwe Blue Frontosa, was due to hypertrophy of the hypodermal layer and increased fat storage (Takahashi 2018).

The nuchal hump is a diagnostic trait for the swordtail species *Xiphophorus birchmanni* (Rauchenberger et al. 1990) and may play a role in mate choice (Rosenthal et al. 2003) as well as influence swimming endurance in relation to the sword (Johnson et al. 2014). Nuchal humps are not known to occur in the wild for any of the other *Xiphophorus* fishes. The development of this trait in the laboratory occurred in *X. multilineatus* adult males that were sampled from a wild population and brought to the laboratory as well as in males that were reared from birth in the laboratory (Tompkins et al. 2021). Females prefer males with humps versus males without (Tompkins et al. 2021), suggesting a potential benefit for the hump via sexual selection if they were to develop in the wild. One aspect of the laboratory environment that promotes the hump is diet, as *X. multilineatus* males reared on high quality diets (i.e., higher fat content in addition to protein) were more likely to develop larger humps than males reared on low quality diets (Tompkins et al. 2021).

Temperature influences fat utilization in fish by stimulating shifts between catabolizing fat as an energy source at warmer acclimation temperatures to storing fat in liver and muscle tissues at colder acclimation temperatures (Stone and Sidell 1981); Egginton and Sidell 1989). Accordingly, we hypothesize that temperature influences the process of hump development. Therefore, to further examine the possible function of the nuchal hump in *X. multilineatus*, we used a split-brood design to examine the development of the hump in the laboratory under two different temperature regimes. We first addressed the following questions: (1) What is the tissue composition of the nuchal hump in *X. multilineatus*? While the presence of lipid droplets has been detected in the nuchal hump of *X. multilineatus* (Tompkins et al. 2021), it is not known if the hump is composed primarily of adipose tissue. (2) Are there differences in the genes expressed in hump tissue in males reared in a cold environment versus warm? (3) How does rearing temperature influence the allometric growth of the nuchal hump? Second, we reduced the diet of adult males for approximately 1 month to ask (4) is the size of the hump influenced by a reduction in diet? (5) Is the influence of diet reduction consistent across temperature treatments? And finally, we examined activity levels to ask (6) is the size of the hump influenced by activity? (7) Is the influence of activity consistent across temperature treatments?

## Methods

### Animals and sample collection

*X. multilineatus* males used to test the effects of rearing temperature on the development of nuchal humps, through both RNA sequencing (RNA-Seq) analyses and multivariate analysis of hump size and growth rate, were obtained by isolating 50 pregnant females from an existing community tank with only courter male *X. multilineatus* located in the Morris Lab. Courter males are one of two alternative reproductive tactics (ARTs) found in this species. ARTs in *X. multilineatus* are genetically influenced by variation in copy number of *mc4r* B alleles on the Y chromosome (Lampert et al. 2010). The ARTs are dimorphic for several traits including body size (courter males are larger than sneakers) and reproductive behavior, where courter males use only courtship displays to attract females and sneaker males use either courtship or forced copulation to gain access to females (Liotta et al. 2021). Courter males in the lab were observed to have larger humps relative to their body size than sneaker males, so only males with a courter lineage were used in this study due to the higher propensity of humps forming on courter males. Females were allowed to drop fry, and the fry were split between two environmental chambers. Environmental chambers were set at 25 °C (warm treatment) and 20 °C (cold treatment). Fry were transferred to individual 2.5-L tanks in respective environmental chambers at 30 days of age: 72 fry in warm and 72 fry in cold. All fry were fed a high-quality diet of Ken’s Spirulina flake food in the morning daily and brine shrimp in the afternoon 5 days a week. The flake food was the same high-quality diet used by Tompkins et al. (2021) that promoted growth of larger humps. Each tank was equipped with a Whisper® Powerfilter that circulated the water and agitated the water surface. Measures of dissolved oxygen saturation were similar between treatments ((*N* = 8 per treatment, cold = 100.8 ± 1.1%; warm = 97.7 ± 1.4%).

### Histology

**Fig. 1:**
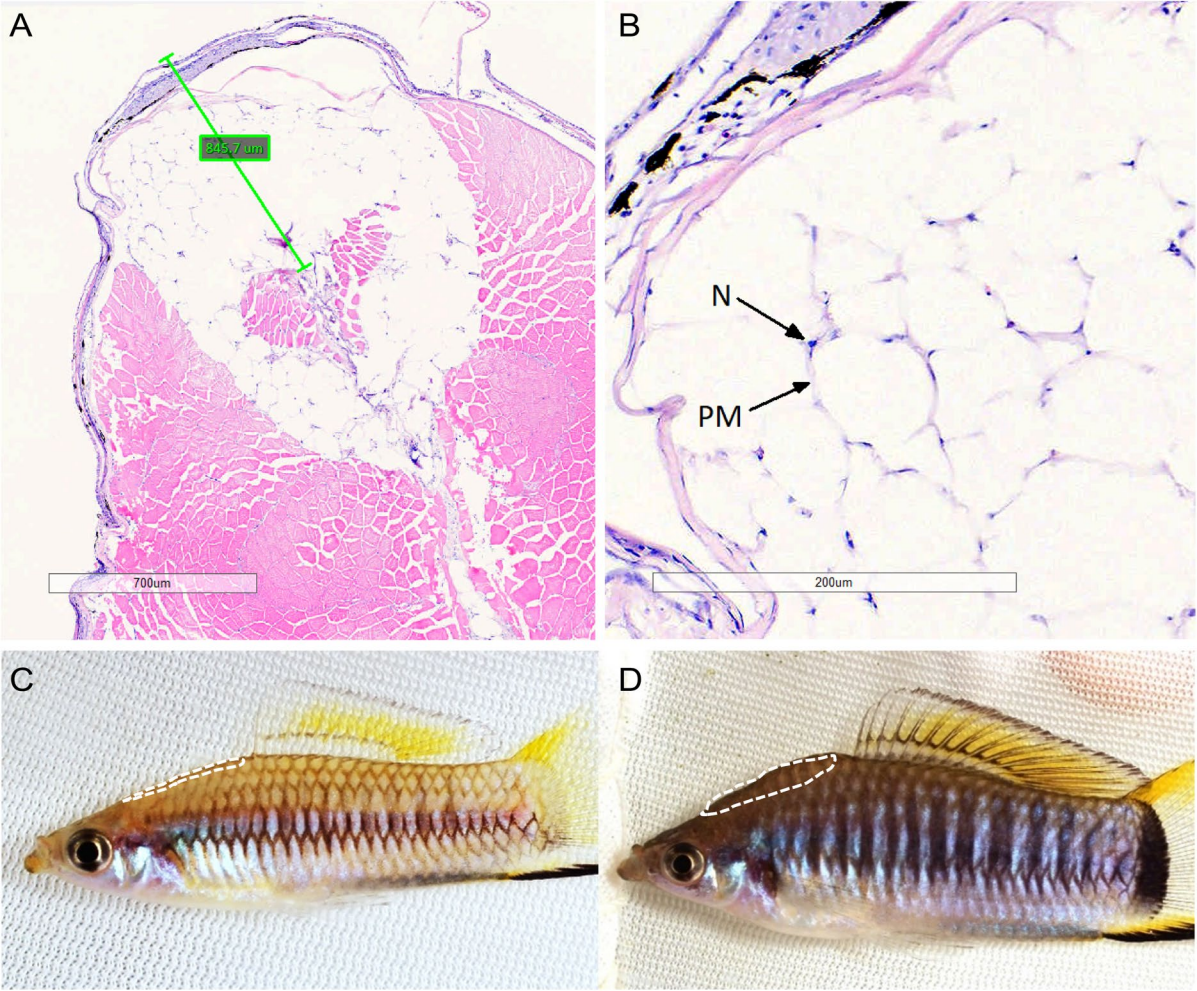
Histology of *X. multilineatus* nuchal hump: **A** transverse section through nuchal hump of male *X. multilineatus*. Bar represents depth of the hump as determined by measuring the fish from a 2D image taken before tissue sampling; **B** adipocytes within nuchal hump (N, nucleus; PM, plasma membrane; H&E); **C** a male from the warm treatment with a relatively small nuchal hump for its size (0.099 mm^2^/mm); and **D** a male from cold treatment with a relatively large hump (0.275 mm^2^/mm). The white dashed lines (**C**, **D**) circumscribe the nuchal regions specifically dissected for nuchal hump tissues

Males used for histology were obtained from a community tank with only *X. multilineatus* located in the Morris Lab. Fish in the community tank were fed a high-quality diet of Ken’s Spirulina® flake food 7 days per week and *Artemia* sp. nauplii 5 days per week at a rate that was completely consumed within 5 min. The community tank was maintained at room temperature between 21 and 22 °C. Males were euthanized with MS-222, and transverse tissue sections were taken from the nuchal region (Fig. 1) and preserved in 10% neutral buffered formalin. Tissue samples were processed and stained at the Ohio University Histology Core Facility using standard eosin and hematoxylin techniques. Stained slides were then digitally scanned, and the depth of the hump was measured and denoted using Aperio ImageScope software. Image J software was used to measure standard length (mm) and nuchal hump area (mm^2^) of the two sections for each fish.

### Gene expression profiling

Three sexually mature males from each temperature treatment were selected for gene expression profiling using RNA sequencing (RNA-Seq) and euthanized with MS-222. Each male had a corresponding full sibling in the opposing dietary treatment. Image J software was used to measure standard length (mm) and nuchal hump area (mm^2^) of each fish. Hump tissue, belly tissues, and livers were dissected from each male (see Fig. 1) and preserved in RNAlater®. RNA was isolated using a method described in an earlier study. Briefly, total RNA from these tissue samples was isolated using TRI-Reagent (Sigma Inc., St. Louis, MO, USA). Tissue samples were homogenized in TRI-Reagent followed by the addition of 200 µl/ml chloroform, and the samples were vigorously shaken and subjected to centrifugation at 12,000 g for 5 min at 4 °C. Total RNA was further purified using the RNeasy mini RNA isolation kit (Qiagen, Valencia, CA, USA). Residual DNA was eliminated by performing column DNase digestion at 25 °C for 15 min. Total RNA concentration was determined using a Qubit 2.0 fluorometer (Life Technologies, Grand Island, NY, USA). RNA quality was verified on an Agilent Bioanalyzer (Agilent Technologies, Santa Clara, CA, USA) to confirm that RIN scores were above 8.0 prior to sequencing.

### Differentially expressed gene analyses

RNA-Seq was performed upon libraries constructed using the Illumina TruSeq library preparation system (Illumina, Inc., San Diego, CA, USA). RNA libraries were sequenced as 150-bp pair-end fragments using the Illumina Novaseq system (Illumina, Inc., San Diego, CA, USA).

Short sequencing reads were mapped to a reference *X. multilineatus* genome using Tophat2 (Reference PMID: 23,618,408). Gene expression profiling was conducted by quantifying sequencing reads that were mapped to exons using the Subread package featureCount function (reference PMID: 24,227,677, 23,558,742). Differentially expressed genes were identified using the R-Bioconductor package edgeR with a statistical cutoff as |Log_2_Fold Change|> 1, false discovery rate < 0.05. Principal component analyses (PCA) were performed using the R prcomp function, using normalized and centered gene expression counts (22287627). Gene Ontology analyses were conducted using g:Profiler (https://biit.cs.ut.ee/gprofiler/gost), using default statistical thresholds.

### Comparison between *X. multilineatus* hump to human adipocyte cell atlas

Three hump tissue samples collected from the cold rearing temperature group were used to compare to genes hallmarking cell types within the human white adipose tissue (PMID: 35,296,864). We used count per million (cpm) > 50 for each sample to determine genes expressed. For each human cell type, we kept genes with false discovery rate (FDR) adjusted *P*-value < 0.05 and compared to hump expressed genes. A hypergeometric test was performed with the null hypothesis that observing a cell type marker in the hump tissue is random. A hypergeometric test *P*-value < 0.05 was used to determine statistical significance.

### Growth and size of the nuchal hump

To determine the effects of temperature on the growth and size of the nuchal hump, fish from temperature treatments were photographed at 30, 100, 210, and 330 days of age to capture nuchal hump developmental rates. The area of the nuchal hump was measured at each age interval using Image J software (Fig. 1). A period of diet restriction was imposed for 1 month, which consisted of only a single daily feeding of flake food. All males placed on a restricted diet were over 400 days old and sexually mature at the time of the restriction. Only full sibling males were used in the analyses to control for genetic effects.

All statistics regarding hump growth and size were calculated using R software. In addition to treatment, we examined whether adult size and early growth rate influenced the development of humps. To determine what factors influenced the size of the adult nuchal humps, a generalized least squares model was used with a random effect of dam. Treatment was the fixed effect with male size at maturity and early growth rate as covariates. The intraclass correlation coefficient (ICC) was calculated to determine the proportion of total variability in hump size that was attributable to differences among dams. The models were run with the restricted maximum likelihood estimation method (REML) to correct for degrees of freedom. Results from performing ANOVA on the model and coefficient estimates for each fixed effect are reported.

Growth curves for nuchal hump size were fitted to the observed data for each treatment using the von Bertalanffy growth function (VBGF), Gompertz (Gomp), and a logistic (Log) curve with the R package “fishmethods” and the function growth. The growth function with the lowest residual sum-of-squares was considered the best fit and was used to calculate asymptotic hump size at maturity (length at which male growth stops) for both treatments. Growth curves were compared by treatment using a pairwise permutation test with the R function compareGrowthRates in the package “fishmethods.”

### Diet restriction and size of nuchal hump

Changes in nuchal hump size before and after a period of diet restriction were compared for each treatment by using a generalized least squares model with a random effect of dam plus a correlation component accounting for repeated measures among males before and after diet restriction. The intraclass correlation coefficient (ICC) was calculated to assess the proportion of total variability in hump size that was attributable to differences among mothers. The degree of correlation between repeated measures per male was estimated by the compound symmetry parameter (rho). Temperature treatment and diet restriction status (either pre- and post-restriction) were used in all models as independent variables, and an interaction term between treatment and diet restriction status was included to determine whether the magnitude of change in hump size after diet restriction was dependent on treatment. Nuchal hump size was log transformed within the model to meet the assumption of normality of the residuals. Results from R function Anova are reported.

### Activity levels and size of the nuchal hump

To test the hypothesis that activity may influence the size of the nuchal hump, we tested the activity levels of 15 males reared in the cold environment and 8 males reared in the warm environment. A test tank was placed in each of the environmental chambers, consisting of a 37.85-l aquarium with gravel on the bottom, marked on the outside with lines dividing it into 12 boxes (Supplemental Fig. S2). After transferring a male to a test tank, he was given 5 min to acclimate. We then measured the number of times an individual crossed into another section of the aquarium over a period of 5 min.

We used a generalized linear model to examine variation in relative nuchal hump size (nuchal hump/SL), considering the factors of temperature treatment, activity levels, and their interaction. The best model was selected based on AIC (difference > 2.0).

## Results

### Histology and gene expression show the hump is adipose tissue

Histological examination shows the hump is primarily composed of adipose tissue (Fig. 1A and B). Stored lipid droplets within adipocytes are cleared during histological processing, leaving only the plasma membrane and peripherally located nucleus visible (Fig. 1B).

**Fig. 2:**
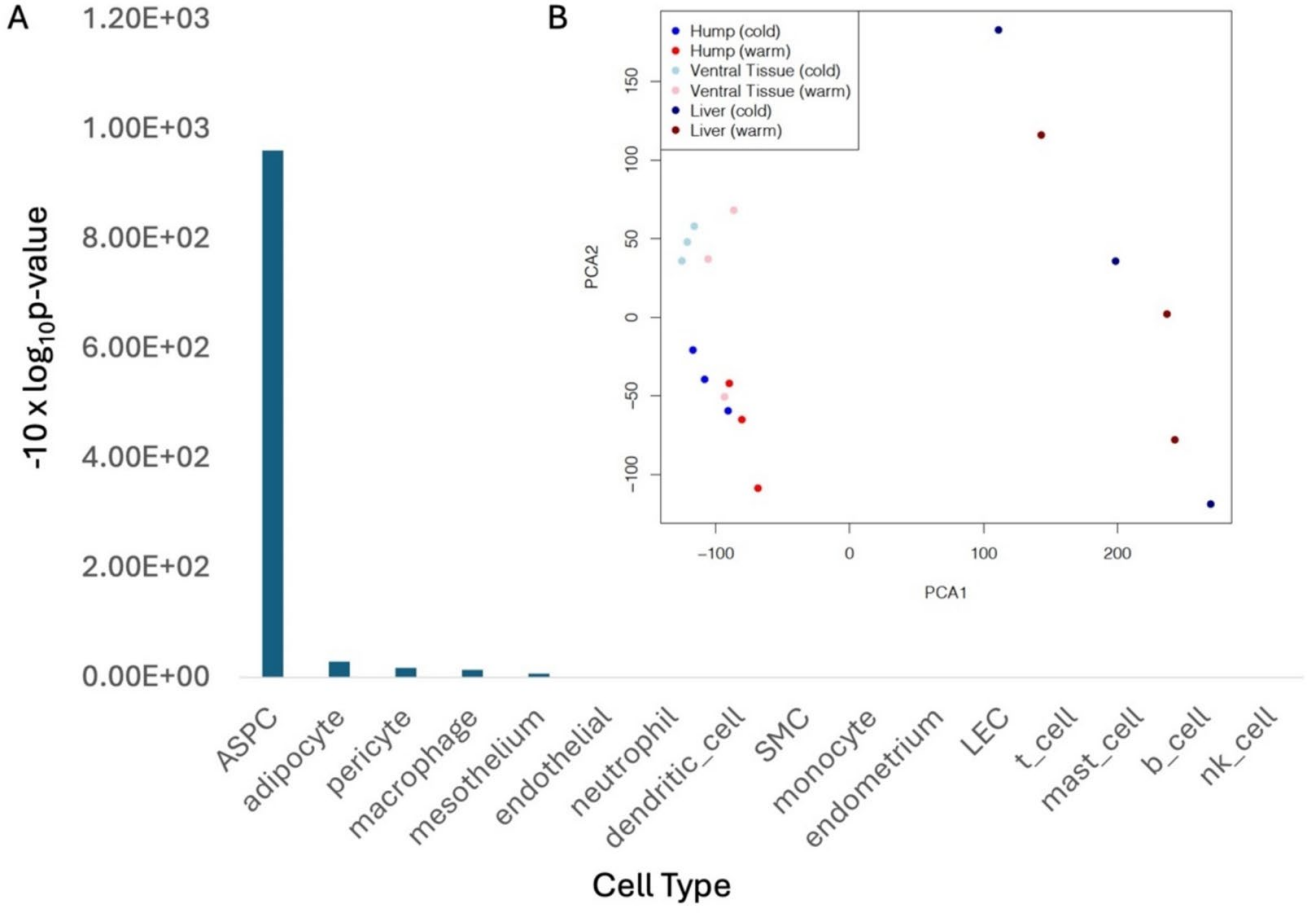
Cell type annotation of *X. multilineatus* hump. **A** Expressed genes in the bulk hump tissue were compared to human white adipocyte cell atlas. The bar heights represent − 10 × log10 hypergeometric test *P*-value. *P*-value < 0.05 is used to determine statistical significance. **B** A dot plot, showing the principal component analyses of *X. multilineatus* liver, belly (ventral), and hump

We compared the nuchal hump gene expression profiles to liver and belly tissue. Principal component analyses showed the hump tissue gene expression pattern is more similar to belly tissue gene expression than to either brain or liver. This is evidenced by the proximity of the hump tissue and belly tissue samples on a dimension-reduced x–y grid (Fig. 2B). These observations suggest that not only the tissue histology but also the molecular phenotype of the hump show they are adipocytes and are likely to be energy-storage tissue. In addition, we compared gene expression in the bulk hump tissue to human cell atlas for white adipocyte (PMID: 35296864) to determine if the adipocyte genes are expressed in the hump. For the 16 cell types annotated in human white adipocyte, gene markers for 4 cell types are found to be over-enriched (*P*-value < 0.05). They are adipose stem and progenitor cells (ASPC), adipocyte, macrophage, and pericyte (Fig. 2A; for gene markers, see Supplemental Table S1 and Fig. S1).

### Gene expression within the hump

Males reared in two different temperatures differentially expressed 326 genes in nuchal hump tissue (|Log_2_FC|> 1; FDR < 0.05). Functional analyses of the differentially expressed genes (DEGs) showed genes involved in development, regulation of cell migration, and regulation of cell communication (Supplemental Table S2). Manual curation of the DEGs showed *Xiphophorus* orthologs to human adipocyte marker genes *hebp2* and *lipe*, and adipose stem and progenitor cell (ASPC) marker genes *cfd*, *tnfaip2*, *kifap3*, *cox6b1*, and *txn* are differentially expressed. In addition, genes that are related to fat deposition and temperature acclimation were identified (Table 1).

**Table 1:** Examples of differentially expressed genes in nuchal hump tissue between temperature treatments known to be related to energy homeostasis. Genes had a false detection rate (FDR) of 0.05

Gene	Related function	Upregulated	Ref
G0S2	Acts as a molecular brake on triglyceride (TG) catabolism	Cold	Heckmann et al. (2012); Yang et al. (2010)
ACP6	An enzyme that regulates lipid metabolism in the mitochondria	Warm	Hiroyama and Takenawa (1999)
HSPA12A	Heat shock protein in the HSP70 family involved in molecular chaperoning and preventing protein denaturing in response to stressors. HSP70 upregulation also inducible by cold shock	Cold	Hu et al. (2022); Reid et al. (2022)
PGC-1α	Highly expressed in brown fat in mammals; strongly induced by cold exposure; linking environmental stimulus to adaptive thermogenesis	Cold	Cheng et al. (2018); Liang and Ward (2006)
WARS-1	Reduction of WARS-1 expression in *C. elegans* increased fat stores and starvation survival	Warm	Webster et al. (2017)
IGFBP3	Insulin-like growth factor binding protein	Cold	Kim (2013)
APOD	Atypical apolipoprotein with broad tissue distribution involved in lipid homeostasis	Cold	Perdomo et al. (2010)
Col7a1	Codes for collagen alpha 1 protein, which occurs in the basement membrane beneath stratified squamous epithelia	Cold	Koca et al. (2023)
APOA1	Structural component of high-density lipoprotein (HDL) or “good” cholesterol in plasma	Cold	Bandarian et al. (2013)
SFRP2	Reported in pre-adipocytes, involved in adipocyte differentiation; secreted by adipose tissue	Cold	Crowley et al. (2016)

### Adult hump size

The model that best explained variation in hump size at 330 days of age (all males sexually mature) included treatment, male size (length) at sexual maturity, and early growth rate with dam as a random effect (Table 2). The intraclass correlation coefficient (ICC) revealed that 35.3% of the total variability in hump size was attributable to variability associated with dam. The negative coefficient for temperature treatment (− 1.01) produced by the model indicates that hump size is predicted to be smaller in the warm treatment than the cold when other fixed variables were held constant. Mean hump size at sexual maturity for males from the warm treatment was 4.60 ± 2.02 mm^2^, while the mean size of humps for males in the cold treatment was 9.62 ± 2.27 mm^2^. The coefficient for male size at maturity (0.54) reveals a positive relationship between hump size and body size, meaning larger males had larger humps when other variables were held constant. The model produced a negative coefficient (− 22.5) for early growth rate indicating a negative relationship with hump size at maturity, meaning males that grew faster as juveniles developed smaller humps by the time they reached sexual maturity compared with slower growing males.

**Table 2:** ANOVA results generated by the best model for explaining variation in nuchal hump size at maturity

Variable	Numerator DF	Denominator DF	*F*-value	*P*-value
(Intercept)	1	24	488.3557	< 0.0001
Treatment	1	24	126.5105	< 0.0001
Size at maturity (L)	1	24	35.9872	< 0.0001
Early growth rate	1	24	5.0096	0.0326

**Fig. 3:**
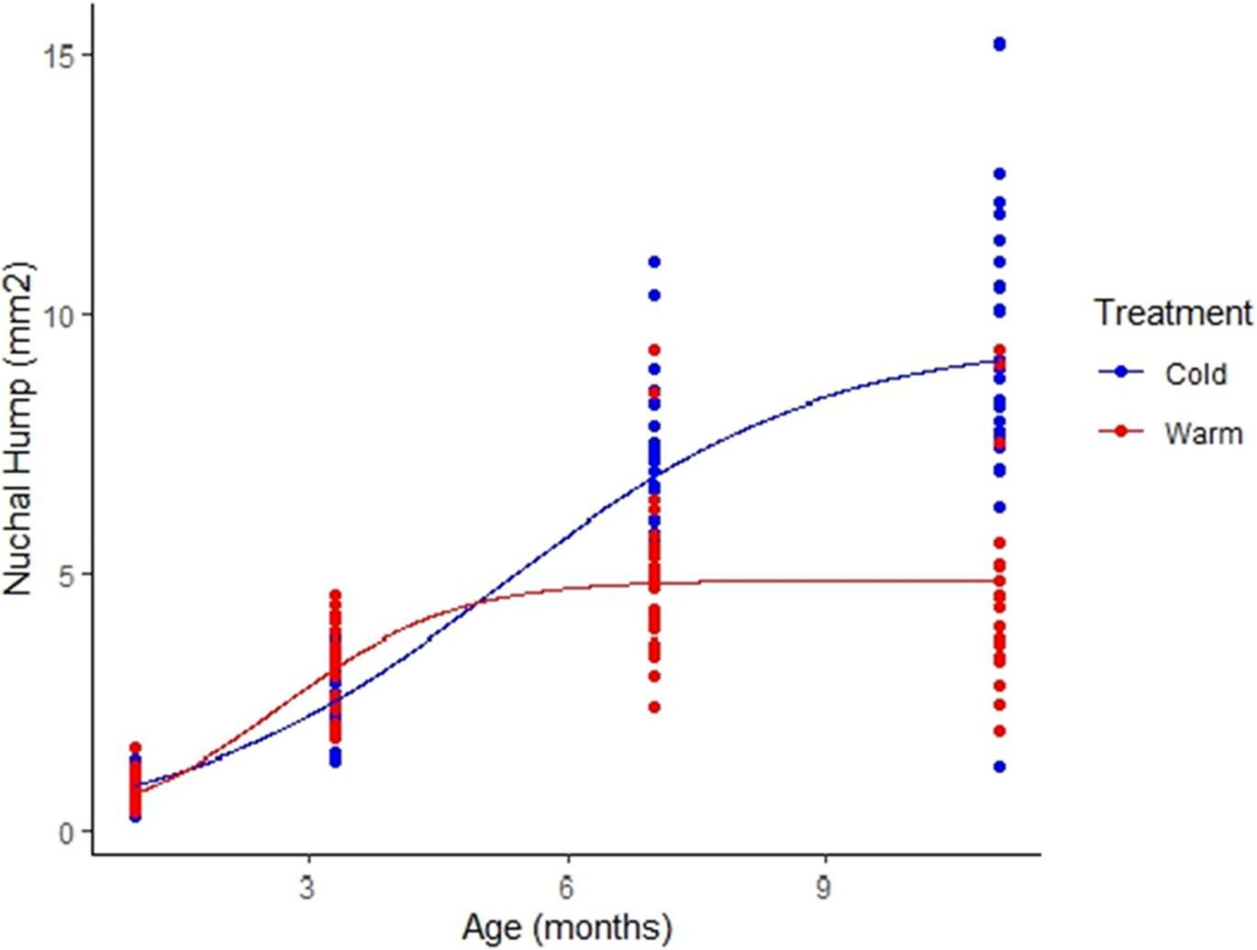
Comparison of nuchal hump growth between temperature treatments. Both growth curves are plotted with a logistic function and were significantly different (*P* = 0.005)

Regarding the growth of the hump, the logistic growth curve had the lowest residual sum of squares and was the best fit compared to the von Bertalanffy and Gompertz for both cold and warm treatments (Table 3). Growth curves for the nuchal hump were significantly different between temperature treatments (*P* = 0.005) and reflect a pattern whereby males from the warm environment grew humps at a faster rate early in development but had smaller humps by the time they reached maturity than males from the cold environment (Fig. 3).

**Table 3:** Nuchal hump growth curve selection data. The logistic (Log) curve had the lowest residual sum of squares (RSS) compared to the von Bertalanffy (VBF) and Gompertz (Gomp) curves in both the *warm* (A) and *cold* (B) treatments

	RSS	*k* ± SE
A) Model		
VBF: Linf*(1-exp(-K*(t-t0)))	217.9	0.55 ± 0.15
Gomp: Linf*exp(-exp(-K*(age-t0)))	214.5	0.77 ± 0.17
Log: Linf/(1 + exp(-K*(age-t0))	212.5	1.12 ± 0.24
B) Model		
VBF: Linf*(1-exp(-K*(t-t0)))	432.5	0.19 ± 0.03
Gomp: Linf*exp(-exp(-K*(age-t0)))	383.1	0.39 ± 0.06
Log: Linf/(1 + exp(-K*(age-t0))	376.9	0.57 ± 0.07

### Diet restriction and size of the nuchal hump

**Fig. 4:**
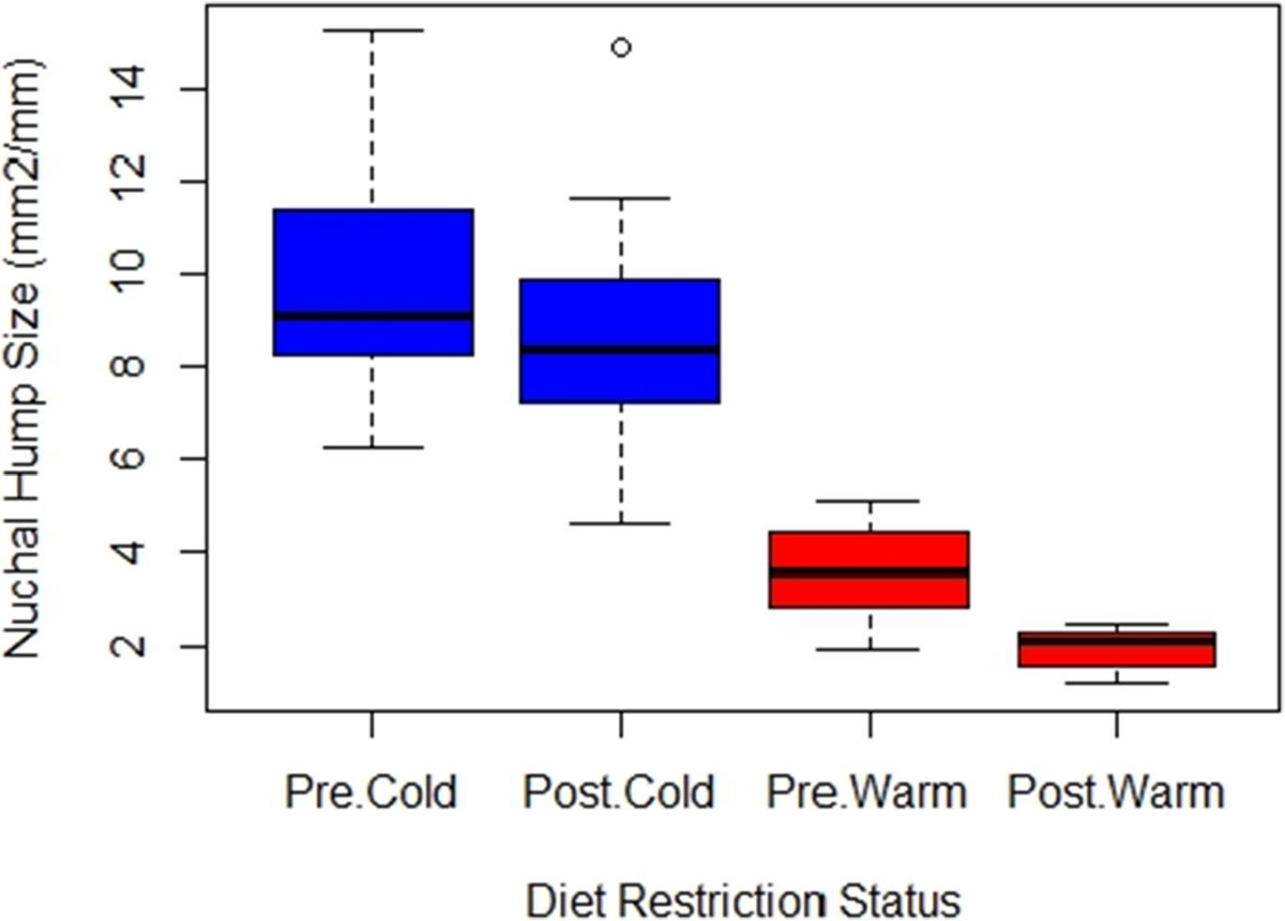
Mean nuchal hump size per treatment before (pre) and after (post) a 1-month period of diet restriction

Following a 1-month period of diet restriction, variation in nuchal hump size was explained best by a model that included treatment, diet restriction status (pre- or post-), and an interaction term between them (Table 4). The model which included dam as a random effect and a correlation component to account for repeated measures was the best fit among the three models compared (*P* < 0.001, Table 4) and variation attributed to dam explained 13.8% of the total variability in hump size in this model as revealed by the intraclass correlation coefficient (ICC). The model produced a high correlation value (rho = 0.805) which suggests that repeated measures for each male are more similar relative to the variability across different males. Negative regression coefficients for treatment (− 1.02) and diet restriction status (− 0.14) indicate that not only were humps estimated to be smaller post-restriction than pre-restriction, but also smaller in the warm treatment versus the cold. More importantly, the coefficient for the interaction term (− 0.45) indicates there is a synergistic effect between treatment and diet restriction, namely that being in the warm environment leads to even smaller post-restriction humps than being in the cold environment. Males from the cold treatment had a mean post-restriction hump size of 8.68 ± 2.58 mm^2^ versus a pre-restriction size of 9.86 ± 2.46 mm^2^, representing a reduction in mean hump size of 1.18 mm^2^ after diet restriction. Males from the warm treatment had a mean post-restriction hump size of 1.97 ± 0.47 mm^2^ versus a pre-restriction size of 3.62 ± 1.06 mm^2^, which is a reduction in mean hump size of 1.64 mm^2^ after diet restriction (Fig. 4).

**Table 4:** ANOVA results generated by the best model for explaining variation in nuchal hump size before and after diet restriction

A	Numerator DF	Denominator DF	*F*-value	*P*-value
(Intercept)	1	33	811.60	< 0.001
Treatment	1	33	127.01	< 0.001
Restriction status	1	33	77.24	< 0.001
*Treatment:restriction status*	1	33	40.69	< 0.001

### Activity levels and size of the nuchal hump

Variation in the relative nuchal hump size (nuchal hump/SL) was best explained by temperature treatment (chi-square = 55.17, df = 1, *P* < 0.001; reared warm vs cold) and activity levels (chi-square = 5.65, df = 1, *P* = 0.017), with higher activity levels correlated with relatively smaller hump size in both treatments. The best model did not include an interaction between these two factors. Overall fitted model compared against the intercept only model (chi-square = 30.417, df = 2, *P* < 0.001) (see Supplemental Fig. S3).

## Discussion

Nuchal hump development in *Xiphophorus multilineatus* males in the laboratory provides a unique opportunity to explore the mechanisms of development and function of this trait. The findings of this study show that the hump consists of adipose tissue, that males reared in a colder environment (20 °C) develop larger humps controlling for overall body size than males reared in a warmer environment (25 °C), and that the differential gene expression pattern in the hump between temperature treatments suggests increased fat deposition in the cold environment versus the warm. Furthermore, changes in the size of the hump due to diet restriction and environmental temperature suggest that the pattern detected is due to increased metabolism in the warm environment stimulating an increased use of fat as an energy source, leading to smaller humps. The implications of these results for understanding the function of the nuchal hump in this system as well as understanding the roles of both diet and activity in human obesity are discussed.

Finding that the nuchal hump is composed primarily of adipocytes suggests an energy storage function of this structure. Certainly, some vertebrates use fat stored in nuchal humps and similar dorsal structures as an energy source when dietary intake is insufficient to maintain metabolic rates, and the amount of fat stored in the hump may reflect seasonal shifts in food quality and quantity (Alexander and Player 1965; Giles et al. 2015; Bengoumi et al. 2005). Within fish taxa, there are examples of nuchal humps composed primarily of adipose tissue, most notably within the species flocks of cichlids that occur in East African rift lakes (Takahashi 2018; Lecaudey et al. 2019). Yet even among this sub-group of fish, energy storage does not appear to be a primary function for humps. Takahashi (2018) found that the nuchal hump of *Cyphotilapia gibberosa* was formed by a thickening of the hypodermis through fat deposition, but the thickness was not correlated with body fitness in females and only slightly positively correlated in males. In other words, well-conditioned individuals did not store extra lipid in the humps, and poorly conditioned individuals did not consume lipid from their humps. In the current study, however, the size of the nuchal hump was smaller after diet restriction regardless of temperature, suggesting that males were able to utilize stored fat in the nuchal hump as an energy source to make up for reduced caloric intake.

If it is assumed that the function of the nuchal hump is for energy storage in *X. multilineatus*, how can the pattern of larger humps on the males reared in the cold versus the warm treatments be explained? Fish acclimated to cold temperatures typically increase their storage of lipids in various body tissues including subcutaneous, liver, and muscle. Fish acclimated to warmer temperatures, on the other hand, increase the use of lipids as a direct energy source and store less of it in tissues (Stone and Sidell 1981); Egginton and Sidell 1989). There is evidence that at extremely high temperatures, fish may shift to storing fat as seen in the mummichog (*Fundulus heteroclitus*, Moerland and Sidell 1986), but fat storage in this case occurred specifically in the liver, not skeletal muscle or hypodermal tissue. The *X. multilineatus* males in the current study were not subjected to critical maximum or minimum temperatures and followed the typical pattern of temperature-related fat utilization in fish. The species of swordtail that forms the nuchal hump in nature, *X. birchmanni*, is found at warmer temperatures on average than a closely related species that does not develop nuchal humps, *X. malinche* (Rauchenberger et al. 1990). These two species often occupy the same stream systems and form hybrid zones (Culumber et al. 2012). When considering the potential influence of temperature alone, hump formation in *X. birchmanni* seems less likely than in *X. malinche*. Therefore, the natural occurrence of nuchal humps in *X. birchmanni* suggests that factors other than temperature are more influential in the development of humps in this species. For example, correlated environmental differences in relation to food resources (higher quality diets) could potentially be more important.

The results of this study suggest that males reared in a warmer environment either utilize more fat than males in the cold or have simply used all the energy available to them with no excess to store in the nuchal humps. The first possibility can be considered the “metabolic difference hypothesis” and is supported by the finding that males from the warm environment used fat stores in the nuchal hump at a faster rate when placed on a restricted diet than males reared in the cold. In addition, seven of the top-tiered, differentially expressed genes in the nuchal hump of *X. multilineatus* males are linked to lipid metabolism (Table 1) with expression patterns suggesting increased fat deposition in cold males versus warm. Overexpression of the *G0S2* switch gene, for example, reduces the rate of lipolysis in adipocytes by acting as a molecular brake on triglyceride catabolism (Yang et al. 2010). A higher expression of G0S2 in *X. multilineatus* males from the cold treatment makes sense since these males developed larger humps. ACP6 that regulates lipid metabolism was upregulated in the warm environment (Hiroyama and Takenawa 1999). Likewise, males from the cold treatment had lower expression of the *WARS-1* gene, which when inactivated in *Caenorhabditis elegans* leads to increased fat stores and increased starvation survival (Webster et al. 2017). Interestingly, a gene that is highly expressed in brown fat in mammals (*PGC-1α*) when induced by cold exposure (Cheng et al. 2018) was also overexpressed in *X. multilineatus* males from the cold treatment. This gene links colder environmental temperature to adaptive thermogenesis by stimulating the catabolic burning of brown fat in mammals; however, fish do not develop brown fat. If the expression pattern of this gene is similar between fish (which are not capable of thermogenesis) and mammals when considering the effect of environmental temperature, its role in thermogenesis among mammals may be a coopted function.

Males from the warm environment grew humps at a faster rate early in development, yet males from the cold developed larger humps by the time they reached sexual maturity (Fig. 3). This is a similar pattern to that of the growth of male body size in general, in that *X. multilineatus* males from the warm environment grew faster as juveniles but reached a smaller adult size than males from the cold (Tompkins et al. in prep). This similarity is not surprising since hump size and body size had a significant positive relationship in the current study.

Tompkins et al. (2021) suggested that diet could be a primary difference between the laboratory and natural environment for *X. multilineatus*, inducing development of the nuchal hump in the laboratory. An additional factor to consider, however, is that the environment in which males were reared (isolated, in 2.5 l aquarium) does not allow for activity levels that would be more common in a natural environment. Swordtail males spend a large proportion of their time interacting with other males and courting females in the wild (Rios-Cardenas et al. 2010), and all these energetically costly behaviors were absent in the laboratory setting. The hypothesis that the reduced activity levels in the rearing environment is partially responsible for the development of the nuchal hump is supported by the observation that *X. multilineatus* males from large mesocosm in our laboratory appear to be much less likely to develop a nuchal hump than males that are reared individually, in addition to the negative relationship we detected between activity levels and relative hump size (Fig. S3). It is also possible that hump size influences activity level due to the hump being a cumbersome feature. However, the shape of *X. birchmanni* with its increased anterior body depth due to having a nuchal hump (the only species in the clade to form them in the wild) imparts improved endurance swimming performance compared with the dorsoventrally narrower shape of the species it naturally hybridizes with, *X. malinche* (Johnson et al. 2014). Therefore, it seems more likely that activity influences hump size rather than vice versa.

There are potential benefits of *X. multilineatus* having the ability to develop a nuchal hump that could be considered. We previously detected female mate preference for males with nuchal humps in *X. multilineatus* even though the trait does not occur in the wild (Tompkins et al. 2021). We suggest that the preference we detected may be similar to what has been suggested for *X. birchmanni* (Rosenthal et al. 2003) and reflect a more general preference for larger males. Alternatively, if the hump developed in *X. multilineatus* in the wild, it could lead to improved swimming performance by increasing anterior body depth, which minimizes drag (Johnson et al. 2014). Compartmentalized fat depots can have dual functionality in other vertebrates. Camels, for example, use the fat in their hump as an energy source, but one hypothesis suggests camels may concentrate body fat in the hump to avoid overheating if the fat was evenly distributed throughout the subcutaneous layer covering the body (Kassa 2016). However, the fact that the humps are not known to form in the natural environments of *X. multilineatus* suggests that selection for these other functions would be minimal if nonexistent. Future studies of the functions of depositing fat in the nuchal hump in swordtail fishes, in addition to energy storage, should be considered.

While nuchal humps can serve many different functions across fishes, the molecular mechanisms involved in their development have been compared to the development of soft tissues on the face of vertebrates in general (Lecaudey et al. 2019). For example, Lecaudey et al. (2021) identified an upstream transcription factor (foxp1) with reduced expression in the hypertrophic lip region of the vacuum cleaner cichlid (*Gnathochromis permaxillaris*), and with a function that has been associated with hypertrophy of the upper lip in humans. In addition, transcription factors with increased expression in the flapped snout of several different species of Lake Malawi cichlids were members of the FOX transcription factor family, with two (foxf1 and foxa2) known to be linked to nose morphogenesis in mammals (Duenser et al. 2023). We are also interested in examining the molecular mechanisms involved in the development of the nuchal hump in swordtail fishes. We plan to use omics approaches, collecting samples for single nucleus RNA sequencing assays of different nuchal hump developmental stages to investigate cellular and molecular pathways leading to the hump.

Evidence that swordtail fish use the nuchal hump for energy storage, with the humps composed of adipose tissue, is interesting considering the importance of identifying evolutionary animal models for studying diet-induced obesity. The development of a nuchal hump in *X. multilineatus* under laboratory conditions, after being fed a high-calorie diet, may be a condition similar to that observed in humans (Schartl and Lu 2024). One of the other aspects of the laboratory environment that appears to promote nuchal hump formation is reduced activity levels, also an important factor in human obesity (Chin et al. 2016). Finally, the influence of environmental temperature on rates of obesity in humans has also been established (Trentinaglia et al. 2021; Moellering and Smith 2012), and cold-induced thermogenesis in mammals is associated with reduced risk of diabetes (Schrauwen et al. 2016; Horino et al. 2022). Considering the influence of temperature, diet, and potentially activity levels on the development of the nuchal hump in *X. multilineatus*, this model system has the potential to provide future insight into the mechanisms involved in human obesity that other model systems cannot. Variation across species within *Xiphophorus* in the propensity to develop nuchal humps allows for the consideration of this trait from an evolutionary perspective, including unique opportunities to determine genetic variant site genotypes using hybrids and backcross hybrids of different species, even from the most phylogenetically distant branches of the genus (Shartl and Lu 2024). By examining the development of the nuchal hump in backcross hybrids of *X. multilineatus* and *X. couchianus*, where we can determine genetic variant site genotypes and profile their gene expression, we plan to disentangle genetic networks involved in fat deposition.

## Supplementary Information

Below is the link to the electronic supplementary material.

Supplementary file1 (PDF 765 KB)

## Data Availability

The data that support the findings of this study are available from the authors upon reasonable request.
