# Supplementary material for: Composition and function of the nuchal hump of male Xiphophorus multilineatus
Source: Fish Physiol Biochem. 2025 Jul 31;51(4):130. doi: 10.1007/s10695-025-01539-2 (PMC12313811; doi:10.1007/s10695-025-01539-2)

# Supporting Information

## Table S1 Results of GO Analysis

GO.Result

| source | term_name | term_id | highlighted | adjusted_p_value | negative_log10_of_adjusted_p_value | term_size | query_size | intersection_size | effective_domain_size | intersections |
|---|---|---|---|---|---|---|---|---|---|---|
| GO:MF | catalytic activity | GO:0003824 | TRUE | 0.013665951 | 1.864360153 | 5751 | 253 | 102 | 20196 | PTPRZ1,PIM1,SARS1,CD38,RDH12,FKBP1B,PAK5,RHOU,RNF19A,CKB,CHIA,PSMB7,OGDH,CSNK2A1,CEL,INPP5J,RAB9A,AANAT,ALOXE3,GLRX,RHOBTB3,ENTPD2,ABCB9,PSAT1,CCL3,PARP14,PLPP7,DHRS1,CYP27B1,CAMK1G,DUSP28,ZC3HAV1,PARP12,ZDHHC2,PLAAT3,F8,PPID,JMJD7,CFD,ARG2,ODC1,ACYP1,MSH4,WARS1,CMPK2,DPP3,MARK4,A3GALT2,HEPHL1,MARS1,RBBP6,RASD1,PDE6H,HS3ST3B1,SUZ12,MMP24,MAGI2,KIF17,ZNRD1,BTD,ENPP2,COX6B1,ENO4,PELI1,AKR1B1,MMP19,PTPN13,EIF2AK2,DOHH,REM2,SRPK3,OBSCN,MYLK4,GAMT,NRDC,CNDP2,PRKCG,VARS1,LIPE,MEP1B,DHDDS,BCO2,DHRS11,ARL14,CEMIP,IDH2,NOCT,IL1R1,CBS,ACP6,CERS6,ACVR1C,PTPRN,ALOX5,TXN,PPA1,CHAC2,MAP3K14,G6PC3,SETD6,FBXL22,AEN |
| GO:BP | system development | GO:0048731 | TRUE | 1.48E-11 | 10.83062634 | 3985 | 250 | 100 | 21017 | PTPRZ1,TFAP2A,PIM1,SARS1,GORASP1,TAGLN3,CD38,FKBP1B,ITSN2,CNTN4,PAPLN,SLC24A4,CKB,TNFAIP2,SLC4A5,OGDH,IL15RA,CCBE1,INPP5J,IGFBP5,RAMP1,CAMSAP3,RHOBTB3,PDGFA,SFRP2,TNC,UNC5D,UNC45B,ADRA2B,XBP1,CCL3,GDF11,CNMD,CXCR4,TAL1,AIRE,APOD,OLFM3,CACNA1C,ZDHHC2,PLAAT3,FOSL1,ARG2,COL10A1,ODC1,MSH4,WARS1,MARK4,NRXN2,SOCS3,HS3ST3B1,TFAP2C,C1QL2,SUZ12,MMP24,SYT1,MAGI2,SLITRK6,BTD,STMND1,COL14A1,ADORA1,COX6B1,MAL,CCDC88A,AKR1B1,GATA3,FHL2,MMP19,PTPN13,VIT,SOX14,AMH,KIFAP3,NRDC,WNT7A,APOH,GRIN2D,PRKCG,FOXO6,FAT3,VTN,APOA1,ATP6AP1,NHS,STRC,KIF26B,RORA,POPDC2,CBS,IRS2,ACVR1C,PTPRN,WNT10A,ALOX5,ZNF219,SLC23A2,ZNF423,IL34,CSPG4 |
| GO:BP | multicellular organismal process | GO:0032501 | FALSE | 8.65E-11 | 10.06273971 | 7234 | 250 | 144 | 21017 | PTPRZ1,CACNA2D4,TFAP2A,PIM1,SARS1,GORASP1,TAGLN3,CD38,RDH12,FKBP1B,PAK5,ITSN2,CNTN4,OTOF,PAPLN,SLC24A4,GPR176,CKB,TNFAIP2,CHIA,DMTN,SLC4A5,OGDH,IL15RA,CCBE1,LMAN1,CEL,INPP5J,STOML2,IGFBP5,RAMP1,ALOXE3,TPPP3,CAMSAP3,RHOBTB3,PDGFA,SFRP2,ENTPD2,TNC,UNC5D,DOCK5,UNC45B,ADRA2B,XBP1,CCL3,GDF15,GDF11,CNMD,G0S2,CYP27B1,CXCR4,TAL1,AIRE,IGFBP3,APOD,OLFM3,ZC3HAV1,CACNA1C,ZDHHC2,COL7A1,PLAAT3,F8,FOSL1,RAB11FIP5,FAT2,ARG2,COL10A1,ODC1,MSH4,WARS1,MARK4,BANF1,NRXN2,RBBP6,SOCS3,PDE6H,HS3ST3B1,TFAP2C,C1QL2,SUZ12,MMP24,SYT1,MAGI2,SLITRK6,GPR137B,BTD,STMND1,COL14A1,ENPP2,ADORA1,COX6B1,PITHD1,PELI1,MAL,CCDC88A,AKR1B1,GATA3,FHL2,MMP19,PTPN13,VIT,EIF2AK2,SOX14,SUCO,AMH,LMOD3,GAMT,KIFAP3,NRDC,WNT7A,APOH,GRIN2D,PRKCG,FOXO6,IGDCC3,FAT3,VTN,SLCO1C1,APOA1,ATP6AP1,CEMIP,NHS,STRC,KIF26B,SYT11,RORA,NOCT,POPDC2,IL1R1,CBS,IRS2,ACVR1C,TRPM2,PTPRN,WNT10A,ALOX5,ZNF219,PPARGC1A,PELO,SLC23A2,SETD6,ZNF423,IL34,CSPG4 |
| GO:BP | multicellular organism | GO:0007275 | FALSE | 1.46E-10 | 9.834202332 | 4658 | 250 | 108 | 21017 | PTPRZ1,TFAP2A,PIM1,SARS1,GORASP1,TAGLN3,CD38,FKBP1B,ITSN2,CNTN4,PAPLN,SLC24A4,CKB,TNFAIP2,SLC4A5,OGDH,IL15RA,CCBE1,INPP5J,IGFBP5,RAMP1,TPPP3,CAMSAP3,RHOBTB3,PDGFA,SFRP2,TNC,UNC5D,UNC45B,ADRA2B,XBP1,CCL3,GDF11,CNMD,CYP27B1,CXCR4,TAL1,AIRE,APOD,OLFM3,CACNA1C,ZDHHC2,COL7A1,PLAAT3,FOSL1,ARG2,COL10A1,ODC1,MSH4 |

| | | | | | | | | | | |
|---|---|---|---|---|---|---|---|---|---|---|
| | development | | | | | | | | | ,WARS1,MARK4,NRXN2,RBBP6,SOCS3,HS3ST3B1,TFAP2C,C1QL2,SUZ12,MMP24,SYT1,MAGI2,SLITRK6,GPR137B,BTD,STMND1,COL14A1,ADORA1,COX6B1,PITHD1,MAL,CCDC88A,AKR1B1,GATA3,FHL2,MMP19,PTPN13,VIT,SOX14,AMH,KIFAP3,NRDC,WNT7A,APOH,GRIN2D,PRKCG,FOXO6,FAT3,VTN,APOA1,ATP6AP1,NHS,STRC,KIF26B,RORA,NOCT,POPDC2,CBS,IRS2,ACVR1C,PTPRN,WNT10A,ALOX5,ZNF219,PELO,SLC23A2,ZNF423,IL34,CSPG4 |
| GO:BP | anatomical structure development | GO:0048856 | FALSE | 1.52E-10 | 9.81931667 | 5924 | 250 | 126 | 21017 | PTPRZ1,TFAP2A,PIM1,SARS1,GORASP1,TAGLN3,CD38,FKBP1B,RHOU,ITSN2,CNTN4,PAPLN,SLC24A4,CKB,TNFAIP2,DMTN,SLC4A5,OGDH,IL15RA,CCBE1,INPP5J,IGFBP5,RAMP1,ALOXE3,TPPP3,CAMSAP3,RHOBTB3,PDGFA,SFRP2,TNC,UNC5D,DOCK5,UNC45B,ADRA2B,XBP1,CCL3,PLPP7,GDF15,GDF11,CNMD,CYP27B1,CXCR4,TAL1,AIRE,IGFBP3,APOD,OLFM3,CACNA1C,ZDHHC2,COL7A1,PLAAT3,FOSL1,ARG2,COL10A1,ODC1,MSH4,WARS1,MARK4,NRXN2,RBBP6,SOCS3,HS3ST3B1,TFAP2C,C1QL2,SUZ12,MMP24,SYT1,MAGI2,SLITRK6,GPR137B,BTD,STMND1,SYCP1,COL14A1,ENPP2,ADORA1,COX6B1,PITHD1,MAL,CCDC88A,AKR1B1,GATA3,FHL2,MMP19,PTPN13,VIT,EIF2AK2,SOX14,AMH,SRPK3,LMOD3,OBSCN,GAMT,KIFAP3,NRDC,WNT7A,APOH,GRIN2D,PRKCG,FOXO6,FAT3,VTN,APOA1,ATP6AP1,NHS,STRC,KIF26B,RORA,NOCT,POPDC2,CBS,IRS2,ACP6,ACVR1C,TRPM2,PTPRN,WNT10A,ALOX5,ZNF219,PPARGC1A,PELO,BICD1,SLC23A2,ZNF423,IL34,CSPG4 |
| GO:BP | developmental process | GO:0032502 | FALSE | 4.08E-09 | 8.389577917 | 6478 | 250 | 130 | 21017 | PTPRZ1,CREM,TFAP2A,PIM1,SARS1,GORASP1,TAGLN3,CD38,FKBP1B,RHOU,ITSN2,CNTN4,SLC22A16,PAPLN,SLC24A4,CKB,TNFAIP2,DMTN,SLC4A5,OGDH,IL15RA,CCBE1,INPP5J,IGFBP5,RAMP1,ALOXE3,TPPP3,CAMSAP3,RHOBTB3,PDGFA,SFRP2,TNC,UNC5D,DOCK5,UNC45B,ADRA2B,XBP1,CCL3,PLPP7,GDF15,GDF11,CNMD,CYP27B1,CXCR4,TAL1,AIRE,IGFBP3,APOD,OLFM3,CACNA1C,ZDHHC2,COL7A1,PLAAT3,FOSL1,ARG2,COL10A1,ODC1,MSH4,WARS1,MARK4,NRXN2,RBBP6,SOCS3,HS3ST3B1,TFAP2C,C1QL2,SUZ12,MMP24,SYT1,MAGI2,SLITRK6,GPR137B,BTD,STMND1,SYCP1,COL14A1,ENPP2,ADORA1,COX6B1,PITHD1,MAL,CCDC88A,AKR1B1,GATA3,FHL2,MMP19,PTPN13,VIT,EIF2AK2,SOX14,SUCO,AMH,SRPK3,LMOD3,OBSCN,GAMT,KIFAP3,NRDC,WNT7A,APOH,GRIN2D,PRKCG,FOXO6,FAT3,VTN,APOA1,ATP6AP1,NHS,STRC,KIF26B,RORA,NOCT,POPDC2,CBS,IRS2,ACP6,ACVR1C,TRPM2,PTPRN,WNT10A,ALOX5,ZNF219,PPARGC1A,PELO,BICD1,SLC23A2,SETD6,ZNF423,IL34,CSPG4 |
| GO:BP | nervous system development | GO:0007399 | FALSE | 1.58244E-05 | 4.800673407 | 2541 | 250 | 63 | 21017 | PTPRZ1,TFAP2A,GORASP1,TAGLN3,CD38,FKBP1B,ITSN2,CNTN4,PAPLN,SLC24A4,CKB,OGDH,IL15RA,INPP5J,CAMSAP3,SFRP2,TNC,UNC5D,ADRA2B,XBP1,CCL3,GDF11,CXCR4,TAL1,APOD,OLFM3,ZDHHC2,MARK4,NRXN2,C1QL2,SUZ12,MMP24,SYT1,MAGI2,SLITRK6,BTD,STMND1,ADORA1,COX6B1,MAL,CCDC88A,GATA3,PTPN13,VIT,SOX14,KIFAP3,NRDC,WNT7A,GRIN2D,PRKCG,FOXO6,FAT3,VTN,STRC,RORA,CBS,IRS2,ACVR1C,WNT10A,SLC23A2,ZNF423,IL34,CSPG4 |
| GO:BP | cell differentiation | GO:0030154 | FALSE | 4.03378E-05 | 4.394287495 | 4381 | 250 | 90 | 21017 | PTPRZ1,CREM,TFAP2A,PIM1,GORASP1,CD38,FKBP1B,ITSN2,CNTN4,SLC22A16,PAPLN,TNFAIP2,DMTN,SLC4A5,OGDH,IL15RA,INPP5J,IGFBP5,ALOXE3,CAMSAP3,SFRP2,TNC,UNC5D,DOCK5,UNC45B,ADRA2B,XBP1,CCL3,PLPP7,GDF15,GDF11,CNMD,CYP27B1,CXCR4,TAL1,AIRE,IGFBP3,APOD,OLFM3,COL7A1,PLAAT3,SOCS3,SUZ12,MMP24,SYT1,MAGI2,SLITRK6,GPR137B,STMND1,SYCP1,COL14A1,PITHD1,MAL,CCDC88A,AKR1B1,GATA3,FHL2,MMP19,EIF2AK2,SOX14,SUCO,AMH,SRPK3,LMOD3,OBSCN,KIFAP3,NRDC,WNT7A,FOXO6,FAT3,VTN,ATP6AP1,NHS,STRC,RORA,NOCT,POPDC2,ACP6,ACVR1C,TRPM2,WNT10A,ALOX5,ZNF219,PPARGC1A,PELO,SLC23A2,SETD6,ZNF423,IL34,CSPG4 |
| GO:BP | cellular developmenta | GO:0048869 | FALSE | 4.07972E-05 | 4.389369358 | 4382 | 250 | 90 | 21017 | PTPRZ1,CREM,TFAP2A,PIM1,GORASP1,CD38,FKBP1B,ITSN2,CNTN4,SLC22A16,PAPLN,TNFAIP2,DMTN,SLC4A5,OGDH,IL15RA,INPP5J,IGFBP5,ALOXE3,CAMSAP3,SFRP2,TNC,UNC5D,DOCK5,UNC45B,ADRA2B,XBP1,CCL3,PLPP7,GDF15,GDF11,CNMD,CYP27B1,CXCR4,TAL1,AIRE,IGFBP3, |

| | | | | | | | | | | |
|---|---|---|---|---|---|---|---|---|---|---|
| | l process | | | | | | | | | APOD,OLFM3,COL7A1,PLAAT3,SOCS3,SUZ12,MMP24,SYT1,MAGI2,SLITRK6,GPR137B,STMND1,SYCP1,COL14A1,PITHD1,MAL,CCDC88A,AKR1B1,GATA3,FHL2,MMP19,EIF2AK2,SOX14,SUCO,AMH,SRPK3,LMOD3,OBSCN,KIFAP3,NRDC,WNT7A,FOXO6,FAT3,VTN,ATP6AP1,NHS,STRC,RORA,NOCT,POPDC2,ACP6,ACVR1C,TRPM2,WNT10A,ALOX5,ZNF219,PPARGC1A,PELO,SLC23A2,SETD6,ZNF423,IL34,CSPG4 |
| GO:BP | anatomical structure morphogenesis | GO:0009653 | FALSE | 5.85969E-05 | 4.232125235 | 2692 | 250 | 64 | 21017 | PTPRZ1,TFAP2A,PIM1,SARS1,GORASP1,RHOU,ITSN2,CNTN4,PAPLN,SLC24A4,TNFAIP2,DMTN,CCBE1,IGFBP5,RAMP1,CAMSAP3,PDGFA,SFRP2,TNC,UNC5D,DOCK5,ADRA2B,XBP1,CCL3,GDF15,GDF11,CNMD,CXCR4,TAL1,AIRE,APOD,OLFM3,CACNA1C,COL7A1,WARS1,SOCS3,HS3ST3B1,SYT1,SLITRK6,ENPP2,GATA3,FHL2,MMP19,SOX14,AMH,LMOD3,OBSCN,GAMT,NRDC,WNT7A,APOH,FAT3,VTN,APOA1,STRC,KIF26B,RORA,CBS,WNT10A,ALOX5,ZNF219,BICD1,SLC23A2,CSPG4 |
| GO:BP | regulation of multicellular organismal process | GO:0051239 | FALSE | 2.55617E-04 | 3.592409454 | 2928 | 250 | 66 | 21017 | PTPRZ1,TFAP2A,PIM1,SARS1,GORASP1,CD38,FKBP1B,SLC24A4,CHIA,DMTN,IL15RA,CCBE1,STOML2,IGFBP5,PDGFA,SFRP2,DOCK5,ADRA2B,XBP1,CCL3,GDF15,CNMD,G0S2,CYP27B1,CXCR4,TAL1,AIRE,APOD,ZC3HAV1,CACNA1C,RAB11FIP5,ARG2,WARS1,BANF1,SLITRK6,GPR137B,COL14A1,ENPP2,ADORA1,PITHD1,PELI1,GATA3,PTPN13,EIF2AK2,SUCO,LMOD3,GAMT,KIFAP3,NRDC,WNT7A,APOH,GRIN2D,VTN,APOA1,SYT11,RORA,NOCT,POPDC2,IL1R1,IRS2,ACVR1C,ALOX5,ZNF219,PPARGC1A,ZNF423,IL34 |
| GO:BP | regulation of developmental process | GO:0050793 | FALSE | 3.88267E-04 | 3.410869901 | 2453 | 250 | 58 | 21017 | PTPRZ1,TFAP2A,PIM1,SARS1,GORASP1,FKBP1B,RHOU,ITSN2,CNTN4,DMTN,IL15RA,CCBE1,IGFBP5,PDGFA,SFRP2,ADRA2B,XBP1,CCL3,PLPP7,GDF15,GDF11,CNMD,CYP27B1,CXCR4,TAL1,IGFBP3,PLAAT3,WARS1,SOCS3,C1QL2,SUZ12,SYT1,SLITRK6,GPR137B,COL14A1,ENPP2,PITHD1,GATA3,PTPN13,EIF2AK2,SUCO,AMH,LMOD3,GAMT,KIFAP3,NRDC,WNT7A,APOH,FOXO6,FAT3,RORA,NOCT,WNT10A,ALOX5,ZNF219,PPARGC1A,SLC23A2,IL34 |
| GO:BP | animal organ development | GO:0048513 | FALSE | 0.001116583 | 2.952108934 | 3047 | 250 | 66 | 21017 | TFAP2A,PIM1,CNTN4,SLC24A4,CKB,SLC4A5,OGDH,CCBE1,IGFBP5,ALOXE3,TPPP3,RHOBTB3,PDGFA,SFRP2,TNC,UNC45B,XBP1,CCL3,GDF11,CNMD,CYP27B1,CXCR4,TAL1,AIRE,APOD,OLFM3,CACNA1C,PLAAT3,FOSL1,ARG2,ODC1,MSH4,RBBP6,SOCS3,HS3ST3B1,TFAP2C,MAGI2,SLITRK6,COL14A1,COX6B1,AKR1B1,GATA3,FHL2,AMH,SRPK3,LMOD3,GAMT,WNT7A,GRIN2D,FAT3,VTN,APOA1,ATP6AP1,NHS,STRC,KIF26B,RORA,POPDC2,CBS,IRS2,PTPRN,WNT10A,ALOX5,ZNF219,PPARGC1A,ZNF423 |
| GO:BP | regulation of cell migration | GO:0030334 | TRUE | 0.001742233 | 2.758893846 | 938 | 250 | 30 | 21017 | DMTN,CCBE1,IGFBP5,CAMSAP3,PDGFA,SFRP2,TNC,UNC5D,DOCK5,XBP1,CCL3,CXCR4,AIRE,IGFBP3,APOD,MIIP,MAGI2,ENPP2,ADORA1,GATA3,SOX14,WNT7A,APOH,VTN,CEMIP,IL1R1,IRS2,ACVR1C,IL34,FRMD5 |
| GO:BP | cell migration | GO:0016477 | FALSE | 0.002459108 | 2.609222367 | 1499 | 250 | 40 | 21017 | PAK5,DMTN,OGDH,CCBE1,IGFBP5,CAMSAP3,PDGFA,SFRP2,TNC,UNC5D,DOCK5,XBP1,CCL3,CXCR4,AIRE,IGFBP3,APOD,GPC6,FAT2,MIIP,MAGI2,ENPP2,ADORA1,CCDC88A,GATA3,SOX14,WNT7A,APOH,FAT3,VTN,APOA1,CEMIP,IL1R1,IRS2,ACVR1C,TRPM2,ALOX5,IL34,FRMD5,CSPG4 |
| GO:BP | neurogenesis | GO:0022008 | FALSE | 0.003085135 | 2.510725843 | 1746 | 250 | 44 | 21017 | PTPRZ1,GORASP1,CD38,FKBP1B,ITSN2,CNTN4,PAPLN,OGDH,IL15RA,INPP5J,CAMSAP3,SFRP2,TNC,UNC5D,ADRA2B,XBP1,CCL3,GDF11,CXCR4,TAL1,APOD,OLFM3,SUZ12,MMP24,SYT1,MAGI2,SLITRK6,STMND1,MAL,CCDC88A,GATA3,SOX14,KIFAP3,NRDC,WNT7A,FOXO6,FAT3,VTN,S |

| | | | | | | | | | | |
|---|---|---|---|---|---|---|---|---|---|---|
| | | | | | | | | | | TRC,RORA,WNT10A,SLC23A2,IL34,CSPG4 |
| GO:BP | regulation of cell motility | GO:2000145 | FALSE | 0.006384455 | 2.194876177 | 1000 | 250 | 30 | 21017 | DMTN,CCBE1,IGFBP5,CAMSAP3,PDGFA,SFRP2,TNC,UNC5D,DOCK5,XBP1,CCL3,CXCR4,AIRE,IGFBP3,APOD,MIIP,MAGI2,ENPP2,ADORA1,GATA3,SOX14,WNT7A,APOH,VTN,CEMIP,IL1R1,IRS2,ACVR1C,IL34,FRMD5 |
| GO:BP | anatomical structure formation involved in morphogenesis | GO:0048646 | FALSE | 0.00905139 | 2.04328474 | 1181 | 250 | 33 | 21017 | TFAP2A,PIM1,SARS1,SLC24A4,TNFAIP2,CCBE1,RAMP1,PDGFA,SFRP2,DOCK5,ADRA2B,XBP1,GDF15,CNMD,CXCR4,TAL1,APOD,COL7A1,WARS1,GATA3,FHL2,MMP19,LMOD3,OBSCN,WNT7A,APOH,FAT3,VTN,KIF26B,RORA,ALOX5,ZNF219,CSPG4 |
| GO:BP | locomotion | GO:0040011 | FALSE | 0.009278617 | 2.032516757 | 1238 | 250 | 34 | 21017 | DMTN,CCBE1,IGFBP5,CAMSAP3,PDGFA,SFRP2,TNC,UNC5D,DOCK5,XBP1,CCL3,CXCR4,AIRE,IGFBP3,APOD,FOSL1,MIIP,MAGI2,ENPP2,ADORA1,GATA3,SOX14,WNT7A,APOH,VTN,APOA1,CEMIP,IL1R1,IRS2,ACVR1C,TRPM2,ALOX5,IL34,FRMD5 |
| GO:BP | regulation of locomotion | GO:0040012 | FALSE | 0.01436215 | 1.842780542 | 1042 | 250 | 30 | 21017 | DMTN,CCBE1,IGFBP5,CAMSAP3,PDGFA,SFRP2,TNC,UNC5D,DOCK5,XBP1,CCL3,CXCR4,AIRE,IGFBP3,APOD,MIIP,MAGI2,ENPP2,ADORA1,GATA3,SOX14,WNT7A,APOH,VTN,CEMIP,IL1R1,IRS2,ACVR1C,IL34,FRMD5 |
| GO:BP | cell motility | GO:0048870 | FALSE | 0.026956998 | 1.569328473 | 1716 | 250 | 41 | 21017 | PAK5,SLC22A16,DMTN,OGDH,CCBE1,IGFBP5,CAMSAP3,PDGFA,SFRP2,TNC,UNC5D,DOCK5,XBP1,CCL3,CXCR4,AIRE,IGFBP3,APOD,GPC6,FAT2,MIIP,MAGI2,ENPP2,ADORA1,CCDC88A,GATA3,SOX14,WNT7A,APOH,FAT3,VTN,APOA1,CEMIP,IL1R1,IRS2,ACVR1C,TRPM2,ALOX5,IL34,FRMD5,CSPG4 |
| GO:BP | positive regulation of cell communication | GO:0010647 | TRUE | 0.029264061 | 1.533665406 | 1783 | 250 | 42 | 21017 | PIM1,CD38,CCBE1,CSNK2A1,IGFBP5,PDGFA,SFRP2,ADRA2B,XBP1,CCL3,PARP14,GDF15,G0S2,CYP27B1,CXCR4,IGFBP3,ZC3HAV1,ZDHHC2,MARK4,PLEKHF1,PDE6H,SYT1,GPR137B,ADORA1,PELI1,MAL,CCDC88A,GATA3,EIF2AK2,AMH,WNT7A,GRIN2D,PRKCG,VTN,APOA1,IL1R1,IRS2,TXN,PELO,ZNF423,IL34,CSPG4 |
| GO:BP | positive regulation of signaling | GO:0023056 | FALSE | 0.029659307 | 1.527839007 | 1784 | 250 | 42 | 21017 | PIM1,CD38,CCBE1,CSNK2A1,IGFBP5,PDGFA,SFRP2,ADRA2B,XBP1,CCL3,PARP14,GDF15,G0S2,CYP27B1,CXCR4,IGFBP3,ZC3HAV1,ZDHHC2,MARK4,PLEKHF1,PDE6H,SYT1,GPR137B,ADORA1,PELI1,MAL,CCDC88A,GATA3,EIF2AK2,AMH,WNT7A,GRIN2D,PRKCG,VTN,APOA1,IL1R1,IRS2,TXN,PELO,ZNF423,IL34,CSPG4 |
| GO:BP | phosphate- | GO:000679 | TRUE | 0.031364972 | 1.503555101 | 2550 | 250 | 54 | 21017 | PTPRZ1,PIM1,CD38,PAK5,CKB,DMTN,OGDH,CSNK2A1,INPP5J,STOML2,PDGFA,SFRP2,ENTPD2,ADRA2B,XBP1,PARP14,CAMK1G,IGFBP3,DUSP28,PLAAT3,ACYP1,WARS1,CMPK2,MARK4,SOC |

| | | | | | | | | | | |
|---|---|---|---|---|---|---|---|---|---|---|
| | containing compound metabolic process | 6 | | | | | | | | S3,MAGI2,ENPP2,ENO4,CCDC88A,PTPN13,EIF2AK2,SRPK3,OBSCN,MYLK4,PRKCG,LIPE,DHDDS,VTN,APOA1,CEMIP,IDH2,RORA,NOCT,IRS2,ACP6,ACVR1C,PTPRN,TXN,PPARGC1A,PPA1,MAP3K14,G6PC3,IL34,CSPG4 |
| GO:BP | phosphorus metabolic process | GO:0006793 | FALSE | 0.03242871 | 1.489070324 | 2553 | 250 | 54 | 21017 | PTPRZ1,PIM1,CD38,PAK5,CKB,DMTN,OGDH,CSNK2A1,INPP5J,STOML2,PDGFA,SFRP2,ENTPD2,ADRA2B,XBP1,PARP14,CAMK1G,IGFBP3,DUSP28,PLAAT3,ACYP1,WARS1,CMPK2,MARK4,SOCS3,MAGI2,ENPP2,ENO4,CCDC88A,PTPN13,EIF2AK2,SRPK3,OBSCN,MYLK4,PRKCG,LIPE,DHDDS,VTN,APOA1,CEMIP,IDH2,RORA,NOCT,IRS2,ACP6,ACVR1C,PTPRN,TXN,PPARGC1A,PPA1,MAP3K14,G6PC3,IL34,CSPG4 |
| GO:BP | negative regulation of multicellular organismal process | GO:0051241 | FALSE | 0.032938397 | 1.48229754 | 1088 | 250 | 30 | 21017 | SARS1,GORASP1,CD38,FKBP1B,IGFBP5,PDGFA,SFRP2,DOCK5,CCL3,GDF15,CNMD,APOD,RAB11FIP5,ARG2,BANF1,GPR137B,ADORA1,PELI1,GATA3,PTPN13,KIFAP3,NRDC,WNT7A,APOH,VTN,APOA1,SYT11,ACVR1C,ALOX5,ZNF423 |
| GO:BP | regulation of signaling | GO:0230051 | FALSE | 0.03845607 | 1.415035105 | 3446 | 250 | 67 | 21017 | PIM1,CD38,FKBP1B,PAK5,ARMH4,RHOU,CNTN4,RNF19A,OTOF,SLC24A4,CCBE1,CSNK2A1,IGFBP5,RAMP1,PDGFA,SFRP2,ADRA2B,XBP1,CCL3,PARP14,GDF15,G0S2,CYP27B1,CXCR4,IGFBP3,APOD,ZC3HAV1,ZDHHC2,RAB11FIP5,GPC6,MARK4,BANF1,GRIA4,PLEKHF1,SOCS3,PDE6H,SYT1,MAGI2,GPR137B,ADORA1,PELI1,MAL,CCDC88A,GATA3,FHL2,PTPN13,EIF2AK2,AMH,OBSCN,WNT7A,GRIN2D,PRKCG,VTN,APOA1,SYT11,RORA,IL1R1,CBS,IRS2,ACVR1C,ALOX5,TXN,PELO,BICD1,ZNF423,IL34,CSPG4 |
| GO:BP | regulation of cell communication | GO:0010646 | FALSE | 0.041099898 | 1.386159254 | 3453 | 250 | 67 | 21017 | PIM1,CD38,FKBP1B,PAK5,ARMH4,RHOU,CNTN4,RNF19A,OTOF,SLC24A4,CCBE1,CSNK2A1,IGFBP5,RAMP1,PDGFA,SFRP2,ADRA2B,XBP1,CCL3,PARP14,GDF15,G0S2,CYP27B1,CXCR4,IGFBP3,APOD,ZC3HAV1,ZDHHC2,RAB11FIP5,GPC6,MARK4,BANF1,GRIA4,PLEKHF1,SOCS3,PDE6H,SYT1,MAGI2,GPR137B,ADORA1,PELI1,MAL,CCDC88A,GATA3,FHL2,PTPN13,EIF2AK2,AMH,OBSCN,WNT7A,GRIN2D,PRKCG,VTN,APOA1,SYT11,RORA,IL1R1,CBS,IRS2,ACVR1C,ALOX5,TXN,PELO,BICD1,ZNF423,IL34,CSPG4 |
| GO:BP | positive regulation of signal transduction | GO:0009967 | FALSE | 0.041553335 | 1.381394119 | 1566 | 250 | 38 | 21017 | PIM1,CCBE1,CSNK2A1,IGFBP5,PDGFA,SFRP2,ADRA2B,XBP1,CCL3,PARP14,GDF15,G0S2,CYP27B1,CXCR4,IGFBP3,ZC3HAV1,MARK4,PLEKHF1,PDE6H,GPR137B,ADORA1,PELI1,MAL,CCDC88A,GATA3,EIF2AK2,AMH,WNT7A,GRIN2D,VTN,APOA1,IL1R1,IRS2,TXN,PELO,ZNF423,IL34,CSPG4 |
| GO:BP | generation of neurons | GO:0048699 | FALSE | 0.048488752 | 1.314358995 | 1517 | 250 | 37 | 21017 | PTPRZ1,GORASP1,CD38,FKBP1B,ITSN2,CNTN4,PAPLN,OGDH,IL15RA,INPP5J,CAMSAP3,SFRP2,TNC,UNC5D,ADRA2B,XBP1,GDF11,CXCR4,TAL1,APOD,OLFM3,SYT1,MAGI2,SLITRK6,STMND1,CCDC88A,GATA3,SOX14,KIFAP3,NRDC,WNT7A,FOXO6,FAT3,STRC,RORA,WNT10A,SLC23A2 |

| | | | | | | | | | | |
|---|---|---|---|---|---|---|---|---|---|---|
| GO:CC | cytoplasm | GO:0005737 | TRUE | 2.0164E-05 | 4.695422625 | 12394 | 256 | 185 | 22115 | CREM,IPO4,PIM1,SARS1,GORASP1,RDH12,RBIS,PSME2,LARP4B,FKBP1B,PAK5,RHOU,ITSN2,RNF19A,SLC22A16,OTOF,SLC24A4,AHSA1,CKB,TNFAIP2,CHIA,PSMB7,DMTN,OGDH,MRPS24,IL15RA,MRC1,LMAN1,CSNK2A1,SVOP,CEL,RUSC2,INPP5J,STOML2,IGFBP5,COL28A1,RAB9A,AANAT,ALOXE3,TPPP3,GLRX,CAMSAP3,RHOBTB3,PDGFA,ENTPD2,TNC,ABCB9,DOCK5,PHYHIP,UNC45B,TMED7,ADRA2B,PSAT1,XBP1,CCL3,FMOD,PARP14,CFAP77,PLPP7,GDF15,G0S2,DHRS1,CYP27B1,CXCR4,CAMK1G,AIRE,IGFBP3,HIGD1A,APOD,OLFM3,APOO,ZC3HAV1,CACNA1C,ZDHHC2,COL7A1,PLAAT3,F8,RAB11FIP5,GPC6,FAT2,PPID,JMJD7,NDUFAF3,CFD,ARG2,COL10A1,ODC1,WARS1,CMPK2,DPP3,MARK4,BANF1,KCNIP1,RTN2,A3GALT2,GRIA4,CLIP2,PLEKHF1,MARS1,RBBP6,MPRIP,PRELID3B,RASD1,SOCS3,HS3ST3B1,TFAP2C,HEBP2,MMP24,SYT1,MAGI2,GPR137B,KIF17,BET1,BTD,STMND1,MLLT11,COL14A1,COX6B1,PITHD1,ENO4,PELI1,MAL,CCDC88A,AKR1B1,FHL2,PTPN13,EIF2AK2,DOHH,SUCO,TMPO,ERICH3,SRPK3,LMOD3,OBSCN,GAMT,KIFAP3,NRDC,CNDP2,WNT7A,APOH,GRIN2D,PRKCG,VARS1,LIPE,DHDDS,FOXO6,BCO2,ARL14,VTN,APOA1,TAPBPL,ATP6AP1,CEMIP,IDH2,NHS,TEX9,PPFIBP2,KIF26B,SYT11,NOCT,WDR4,CBS,IRS2,ACP6,CERS6,TRPM2,PTPRN,HSPA12A,EPB41L1,ALOX5,TXN,PPARGC1A,PELO,PPA1,CHAC2,MAP3K14,G6PC3,BICD1,SLC23A2,NUTF2,SETD6,MYO5A,FBXL22,ANKRD34B,CSPG4 |
| GO:CC | extracellular region | GO:0005576 | TRUE | 9.70025E-05 | 4.013216952 | 4232 | 256 | 83 | 22115 | PTPRZ1,SARS1,CD38,PSME2,ITSN2,CNTN4,PAPLN,AHSA1,CKB,TNFAIP2,CHIA,PSMB7,IL15RA,CCBE1,LMAN1,CEL,RUSC2,IGFBP5,COL28A1,RAB9A,GLRX,RHOBTB3,PDGFA,SFRP2,ENTPD2,TNC,PSAT1,CCL3,FMOD,GDF15,GDF11,CNMD,CXCR4,PRG4,IGFBP3,APOD,OLFM3,APOO,COL7A1,F8,GPC6,FAT2,CFD,COL10A1,WARS1,HHIPL1,DPP3,GRIA4,MARS1,HEBP2,C1QL2,MMP24,BTD,COL14A1,ENPP2,AKR1B1,MMP19,PTPN13,ELOA,VIT,AMH,OBSCN,CNDP2,WNT7A,APOH,MEP1B,DHRS11,VTN,APOA1,ATP6AP1,CEMIP,IDH2,PPFIBP2,IL1R1,WNT10A,HSPA12A,ALOX5,TXN,PPA1,NUTF2,IL34,MYO5A,CSPG4 |
| GO:CC | extracellular matrix | GO:0031012 | TRUE | 2.19687E-04 | 3.658196528 | 555 | 256 | 22 | 22115 | PTPRZ1,CCBE1,LMAN1,COL28A1,SFRP2,ENTPD2,TNC,FMOD,GDF15,PRG4,COL7A1,GPC6,COL10A1,MMP24,COL14A1,MMP19,VIT,WNT7A,APOH,VTN,APOA1,CSPG4 |
| GO:CC | external encapsulating structure | GO:0030312 | FALSE | 2.26397E-04 | 3.64512852 | 556 | 256 | 22 | 22115 | PTPRZ1,CCBE1,LMAN1,COL28A1,SFRP2,ENTPD2,TNC,FMOD,GDF15,PRG4,COL7A1,GPC6,COL10A1,MMP24,COL14A1,MMP19,VIT,WNT7A,APOH,VTN,APOA1,CSPG4 |
| GO:CC | extracellular space | GO:0005615 | FALSE | 3.36178E-04 | 3.473430075 | 3303 | 256 | 68 | 22115 | SARS1,CD38,PSME2,ITSN2,AHSA1,CKB,TNFAIP2,CHIA,CCBE1,LMAN1,CEL,RUSC2,IGFBP5,COL28A1,RAB9A,GLRX,RHOBTB3,PDGFA,SFRP2,ENTPD2,TNC,PSAT1,CCL3,FMOD,GDF15,GDF11,CXCR4,PRG4,IGFBP3,APOD,OLFM3,APOO,COL7A1,F8,FAT2,CFD,COL10A1,WARS1,DPP3,MARS1,HEBP2,MMP24,BTD,COL14A1,ENPP2,AKR1B1,MMP19,PTPN13,ELOA,AMH,OBSCN,CNDP2,WNT7A,APOH,VTN,APOA1,ATP6AP1,IDH2,PPFIBP2,WNT10A,HSPA12A,ALOX5,TXN,PPA1,NUTF2,IL34,MYO5A,CSPG4 |
| GO:CC | cell periphery | GO:0071944 | FALSE | 9.10553E-04 | 3.040694732 | 6269 | 256 | 107 | 22115 | PTPRZ1,CACNA2D4,PIM1,CD38,RDH12,PAK5,RHOU,ITSN2,CNTN4,SLC22A16,OTOF,PAPLN,SLC24A4,GPR176,CKB,TNFAIP2,DMTN,SLC4A5,IL15RA,MRC1,CCBE1,LMAN1,CSNK2A1,SVOP,INPP5J,STOML2,RAMP1,UNC80,COL28A1,RAB9A,SFRP2,ENTPD2,TNC,UNC5D,DOCK5,ADRA2B,FMOD,PARP14,GDF15,CXCR4,PRG4,CAMK1G,OLFM3,CACNA1C,ZDHHC2,COL7A1,PLAAT3,F8,SLC43A3,FOSL1,GPC6,FAT2,COL10A1,KCNIP1,RTN2,NRXN2,HEPHL1,GRIA4,CLIP2,RASD1,SOCS3,PDE6H,HS3ST3B1,MMP24,SYT1,MAGI2,SLITRK6,GPR137B,KIF17,COL14A1,ENPP2,ADORA1,SLC2 |

| | | | | | | | | | | |
|---|---|---|---|---|---|---|---|---|---|---|
| | | | | | | | | | | A9,MAL,CCDC88A,MMP19,PTPN13,VIT,REM2,OBSCN,KIFAP3,WNT7A,APOH,GRIN2D,PRKCG,LIPE,MEP1B,IGDCC3,FAT3,VTN,SLCO1C1,APOA1,TAPBPL,ATP6AP1,TMC3,CEMIP,NHS,SYT11,POPDC2,IL1R1,IRS2,ACVR1C,TRPM2,PTPRN,EPB41L1,SLC23A2,CSPG4 |
| GO:CC | collagen-containing extracellular matrix | GO:0062023 | FALSE | 9.61296E-04 | 3.017142903 | 425 | 256 | 18 | 22115 | PTPRZ1,LMAN1,COL28A1,SFRP2,ENTPD2,TNC,FMOD,GDF15,PRG4,COL7A1,GPC6,COL10A1,COL14A1,VIT,APOH,VTN,APOA1,CSPG4 |
| GO:CC | endoplasmic reticulum-Golgi intermediate compartment membrane | GO:0033116 | TRUE | 0.01605545 | 1.794377526 | 81 | 256 | 7 | 22115 | GORASP1,LMAN1,TMED7,COL7A1,F8,BET1,ATP6AP1 |
| GO:CC | endoplasmic reticulum | GO:0005783 | FALSE | 0.020292813 | 1.692657756 | 2066 | 256 | 44 | 22115 | RDH12,FKBP1B,OTOF,AHSA1,IL15RA,LMAN1,IGFBP5,COL28A1,RAB9A,PDGFA,ENTPD2,TNC,ABCB9,TMED7,XBP1,PLPP7,DHRS1,IGFBP3,APOD,APOO,ZDHHC2,COL7A1,PLAAT3,F8,COL10A1,RTN2,RASD1,BET1,COL14A1,MAL,CCDC88A,SUCO,KIFAP3,WNT7A,GRIN2D,DHDDS,VTN,APOA1,TAPBPL,ATP6AP1,CEMIP,CERS6,G6PC3,MYO5A |
| GO:CC | growth factor complex | GO:0036454 | TRUE | 0.030688175 | 1.513028937 | 8 | 256 | 3 | 22115 | IGFBP5,PDGFA,IGFBP3 |
| REAC | Cytosolic tRNA aminoacylation | REAC:R-HSA-379716 | FALSE | 0.013400036 | 1.872894024 | 24 | 172 | 5 | 10916 | SARS1,WARS1,MARS1,VARS1,PPA1 |
| HPA | skin 1; cells in basal layer[≥Low] | HPA:0461391 | FALSE | 0.003800815 | 2.420123223 | 881 | 156 | 29 | 11019 | TFAP2A,TNFAIP2,PSMB7,LMAN1,STOML2,RAB9A,TMED7,PSAT1,PARP14,APOD,APOO,FAT2,NDUFAF3,BANF1,MPRIP,TFAP2C,ZNRD1,COX6B1,AKR1B1,GATA3,FHL2,TMPO,KIFAP3,CNDP2,IDH2,MPHOSPH10,PPFIBP2,TXN,PPA1 |
| HPA | skin 2; cells in basal | HPA:0471391 | FALSE | 0.01923406 | 1.715929028 | 962 | 156 | 29 | 11019 | TFAP2A,TNFAIP2,PSMB7,LMAN1,STOML2,RAB9A,TPPP3,TMED7,PSAT1,PARP14,APOD,APOO,FAT2,NDUFAF3,BANF1,MPRIP,TFAP2C,ZNRD1,COX6B1,AKR1B1,FHL2,TMPO,KIFAP3,CNDP2,IDH2,MPHOSPH10,PPFIBP2,TXN,PPA1 |

| | | | | | | | | | | |
|---|---|---|---|---|---|---|---|---|---|---|
| | layer[≥ Low] | | | | | | | | | |

**Figure S1** Gene markers

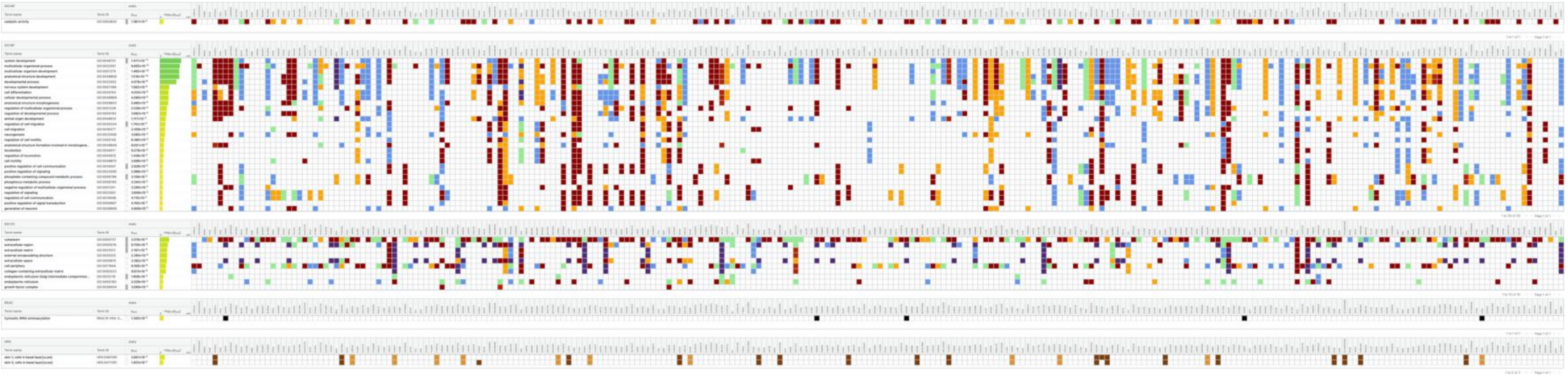

**Table S2** List of all differentially expressed genes in the nuchal hump, between warm and cold treatment males.

| ID | logFC | logCPM | PValue | FDR | GeneName | Description |
|---|---|---|---|---|---|---|
| g10020.t1 | 1.75600717 | 3.77656449 | 0.00049301 | 0.04091225 | Ptprz1 | receptor-type tyrosine-protein phosphatase zeta isoform X2 [Xiphophorus maculatus] |
| g10064.t1 | 2.27519398 | 0.99596647 | 0.00028112 | 0.02955679 | CACNA2D4 | voltage-dependent calcium channel subunit alpha-2/delta-4-like isoform X2 [Xiphophoru [illegible] |
| g10172.t1 | -2.4127332 | 2.8606356 | 4.54E-07 | 0.00039317 | CREM | cyclic AMP-responsive element-binding protein 1-like [Xiphophorus couchianus] |
| g10243.t1 | -1.6743427 | 5.8733369 | 4.48E-05 | 0.00933835 | IPO4 | importin-4 [Xiphophorus maculatus] |
| g10254.t1 | 1.52301838 | 4.56740311 | 6.65E-05 | 0.01171157 | TFAP2A | transcription factor AP-2-alpha isoform X1 [Xiphophorus maculatus] |
| g1030.t1 | -1.2687214 | 5.52999225 | 0.00066828 | 0.04714908 | Pim1 | serine/threonine-protein kinase pim-1-like [Xiphophorus maculatus] |
| g1036.t1 | -1.559971 | 7.93905326 | 0.00030663 | 0.0305763 | sars1 | PREDICTED: uncharacterized protein LOC103467056 [Poecilia reticulata] |
| g10368.t1 | -1.6776209 | 3.94567265 | 6.94E-06 | 0.00288157 | Gorasp1 | Golgi reassembly-stacking protein 1-like isoform X1 [Xiphophorus maculatus] |
| g10419.t1 | 6.57623746 | -1.6316661 | 1.55E-05 | 0.00452321 | TAGLN3 | transgelin-3 [Xiphophorus maculatus] |
| g10559.t1 | 3.6009485 | -0.4569455 | 2.01E-05 | 0.0052547 | Cd38 | ADP-ribosyl cyclase/cyclic ADP-ribose hydrolase 1-like isoform X1 [Xiphophorus maculatu [illegible] |
| g10573.t1 | -1.3795488 | 3.18575707 | 0.00049574 | 0.04098221 | RDH12 | retinol dehydrogenase 12-like [Xiphophorus couchianus] |
| g10575.t1 | -1.5257696 | 3.9104142 | 5.84E-05 | 0.01089624 | RBIS | PREDICTED: uncharacterized protein C8orf59 homolog [Poecilia reticulata] |
| g10592.t1 | 1.55231818 | 2.48636972 | 0.00031099 | 0.03075698 | - | uncharacterized protein LOC102232627 [Xiphophorus maculatus] |
| g10624.t1 | -1.5046586 | 5.62080506 | 5.50E-05 | 0.01053883 | PSME2 | proteasome activator complex subunit 2 [Xiphophorus hellerii] |
| g10639.t1 | -1.5300913 | 5.92904812 | 0.00043544 | 0.03772453 | LARP4B | uncharacterized protein LOC116712363 [Xiphophorus hellerii] |
| g10702.t1 | 1.55347174 | 2.57477599 | 0.00013158 | 0.01887274 | ACT7 | uncharacterized protein LOC102223850 isoform X1 [Xiphophorus maculatus] |
| g10830.t1 | -1.8404533 | 3.49305291 | 0.00032031 | 0.03153436 | - | uncharacterized protein LOC114158645 isoform X1 [Xiphophorus couchianus] |
| g10837.t1 | -1.7975349 | 4.78649388 | 0.00035928 | 0.03383301 | FKBP1B | peptidyl-prolyl cis-trans isomerase FKBP1B isoform X1 [Xiphophorus maculatus] |
| g10857.t1 | -2.1116008 | 3.69123261 | 9.66E-06 | 0.00354754 | Pak5 | serine/threonine-protein kinase PAK 5 isoform X2 [Xiphophorus hellerii] |
| g10873.t1 | -1.6396693 | 3.26830175 | 0.00027248 | 0.02919291 | ARMH4 | armadillo-like helical domain-containing protein 4 isoform X1 [Xiphophorus hellerii] |
| g10900.t1 | 1.60177383 | 2.55891023 | 0.00057562 | 0.04365748 | RHOU | rho-related GTP-binding protein RhoV [Xiphophorus maculatus] |
| g10925.t1 | -1.8010658 | 3.29263876 | 1.42E-06 | 0.00093132 | - | uncharacterized protein LOC114159147 [Xiphophorus couchianus] |
| g10964.t1 | 1.23844738 | 3.31456547 | 0.00066943 | 0.04714908 | ITSN2 | intersectin-2-like isoform X1 [Xiphophorus maculatus] |
| g1098.t1 | 7.34040941 | -1.0258825 | 2.48E-07 | 0.00029395 | Cntn4 | contactin-4-like isoform X1 [Xiphophorus hellerii] |
| g11002.t1 | 1.60896765 | 2.61933639 | 0.00029016 | 0.02995459 | RNF19A | E3 ubiquitin-protein ligase RNF19A-like isoform X1 [Xiphophorus hellerii] |

| | | | | | | |
|---|---|---|---|---|---|---|
| g11077.t1 | -3.0414466 | 2.96690033 | 0.00015462 | 0.02067222 | slc22a16 | solute carrier family 22 member 16 [Xiphophorus couchianus] |
| g11117.t1 | 3.13629142 | -0.196474 | 8.22E-05 | 0.01348051 | otof | otoferlin isoform X1 [Xiphophorus couchianus] |
| g11124.t1 | 1.60712047 | 4.19137572 | 0.00029456 | 0.02995459 | Papln | papilin-like [Xiphophorus hellerii] |
| g11207.t1 | 3.05359734 | 0.50524445 | 0.00010548 | 0.01586511 | Slc24a4 | sodium/potassium/calcium exchanger 4 isoform X1 [Xiphophorus maculatus] |
| g11241.t1 | 5.88275344 | -2.1256913 | 0.00058158 | 0.04382031 | Gpr176 | G-protein coupled receptor 176 [Xiphophorus hellerii] |
| g11317.t1 | -1.5402297 | 4.42849925 | 0.0005311 | 0.04244673 | AHSA1 | activator of 90 kDa heat shock protein ATPase homolog 1-like [Xiphophorus hellerii] |
| g11321.t1 | 1.32420353 | 3.5994328 | 0.00019057 | 0.02385865 | CKB | creatine kinase B-type [Xiphophorus maculatus] |
| g11324.t1 | 1.39242632 | 2.51170757 | 0.00038003 | 0.03443953 | Tnfaip2 | tumor necrosis factor alpha-induced protein 2-like isoform X1 [Xiphophorus couchianus] |
| g11430.t1 | -1.5097283 | 3.66273156 | 0.00025291 | 0.02819525 | RGD1562218 | uncharacterized protein C1orf131 homolog [Xiphophorus hellerii] |
| g1144.t1 | -9.4518534 | 0.75497733 | 0.00013326 | 0.01898905 | CHIA | acidic mammalian chitinase-like [Xiphophorus couchianus] |
| g11482.t1 | -1.5853953 | 7.12123762 | 0.0007261 | 0.04896628 | Psmb7 | proteasome subunit beta type-7 isoform X2 [Xiphophorus couchianus] |
| g11512.t1 | 2.45191609 | 1.42135051 | 4.25E-06 | 0.00214177 | DMTN | dematin-like isoform X1 [Xiphophorus hellerii] |
| g11550.t1 | 2.11027737 | 1.0970604 | 4.42E-05 | 0.00928406 | Slc4a5 | electrogenic sodium bicarbonate cotransporter 4-like isoform X3 [Xiphophorus couchianu |
| g11559.t1 | 3.78988537 | 0.67684828 | 0.00022974 | 0.02719048 | ogdh | 2-oxoglutarate dehydrogenase, mitochondrial isoform X1 [Xiphophorus maculatus] |
| g1156.t1 | -1.5954905 | 3.54490832 | 2.53E-05 | 0.00601143 | zgc:112255 | uncharacterized protein C1orf50 homolog [Xiphophorus maculatus] |
| g11562.t1 | -1.5657671 | 5.132907 | 0.00025515 | 0.02819525 | mrps24 | 28S ribosomal protein S24, mitochondrial [Xiphophorus hellerii] |
| g11603.t1 | -1.4248646 | 2.62128823 | 0.00050504 | 0.04115513 | Il15ra | uncharacterized protein LOC116725039 isoform X3 [Xiphophorus hellerii] |
| g11798.t1 | -3.954466 | 0.69903524 | 0.00019476 | 0.0241046 | MRC1 | macrophage mannose receptor 1-like [Xiphophorus maculatus] |
| g11807.t1 | 1.68749442 | 3.35421061 | 0.00067048 | 0.04714908 | ccbe1 | collagen and calcium-binding EGF domain-containing protein 1 isoform X1 [Xiphophorus |
| g11808.t1 | -1.4117637 | 5.41635549 | 0.00028106 | 0.02955679 | LMAN1 | protein ERGIC-53 [Xiphophorus maculatus] |
| g11819.t1 | -1.2737583 | 4.3189158 | 0.00063762 | 0.04588137 | BRIX1 | ribosome biogenesis protein BRX1 homolog [Xiphophorus maculatus] |
| g1186.t1 | -1.5470854 | 4.53129205 | 0.00043045 | 0.03772453 | csnk2a1 | casein kinase II subunit alpha-like [Xiphophorus maculatus] |
| g11931.t1 | 2.31004135 | 0.23436427 | 0.00034538 | 0.0328093 | svop | synaptic vesicle 2-related protein-like [Xiphophorus hellerii] |
| g11944.t1 | 1.40410348 | 6.29991884 | 0.00045596 | 0.03842684 | setd1ba | histone-lysine N-methyltransferase SETD1B isoform X1 [Xiphophorus hellerii] |
| g11962.t1 | 1.57201303 | 3.39235816 | 1.78E-05 | 0.00489846 | - | zinc-binding protein A33-like [Xiphophorus maculatus] |
| g11980.t1 | -1.5793174 | 3.71248424 | 0.00012361 | 0.01796775 | daf-36 | cholesterol 7-desaturase-like [Xiphophorus hellerii] |
| g11981.t1 | -7.458828 | -0.975954 | 0.00055009 | 0.0430951 | CEL | bile salt-activated lipase-like [Xiphophorus couchianus] |
| g12081.t1 | -1.5819762 | 3.46445602 | 0.00070755 | 0.0488052 | inip | SOSS complex subunit C [Xiphophorus maculatus] |
| g12133.t1 | 3.092996 | 0.34911563 | 8.74E-05 | 0.01382297 | Rusc2 | iporin-like isoform X2 [Xiphophorus hellerii] |

| | | | | | | |
|---|---|---|---|---|---|---|
| g12157.t1 | -3.0388035 | 2.27148037 | 1.07E-05 | 0.00366959 | INPP5J | phosphatidylinositol 4,5-bisphosphate 5-phosphatase A [Xiphophorus maculatus] |
| g12195.t1 | -1.1793461 | 4.3680944 | 0.00056091 | 0.0433882 | Stoml2 | stomatin-like protein 2, mitochondrial [Xiphophorus hellerii] |
| g12221.t1 | 2.16326229 | 8.11027687 | 1.29E-05 | 0.00422839 | - | uncharacterized protein LOC116725033 [Xiphophorus hellerii] |
| g12238.t1 | -1.610931 | 6.14824608 | 7.06E-05 | 0.01223946 | Nars | asparagine--tRNA ligase, cytoplasmic isoform X1 [Xiphophorus maculatus] |
| g12298.t1 | -1.7023015 | 3.25311401 | 0.00030064 | 0.03042754 | IGFBP5 | insulin-like growth factor-binding protein 5 [Xiphophorus couchianus] |
| g12305.t1 | -1.774745 | 3.91569043 | 0.00037183 | 0.03439698 | RAMP1 | receptor activity-modifying protein 1-like [Xiphophorus couchianus] |
| g12315.t1 | 1.89458242 | 2.72733764 | 0.00043403 | 0.03772453 | UNC80 | protein unc-80 homolog isoform X2 [Xiphophorus couchianus] |
| g12381.t1 | 1.5894828 | 3.63596796 | 0.00019263 | 0.02397753 | COL28A1 | collagen alpha-1(XXVIII) chain-like [Xiphophorus maculatus] |
| g12418.t1 | -2.1202984 | 2.07292135 | 0.00014088 | 0.01978207 | RAB9A | ras-related protein Rab-9A-like [Xiphophorus hellerii] |
| g12554.t1 | 1.47490161 | 3.06339937 | 0.00061329 | 0.04518119 | - | skin secretory protein xP2-like isoform X1 [Xiphophorus hellerii] |
| g12705.t1 | 2.6274919 | -0.4518136 | 0.00073023 | 0.04896628 | AANAT | serotonin N-acetyltransferase [Xiphophorus maculatus] |
| g12715.t1 | -4.4423666 | 3.10126512 | 0.00016762 | 0.02200302 | ALOXE3 | hydroperoxide isomerase ALOXE3-like [Xiphophorus couchianus] |
| g12728.t1 | -1.5363733 | 4.5236203 | 1.24E-05 | 0.0041208 | - | lysozyme g-like isoform X1 [Xiphophorus hellerii] |
| g13317.t1 | -7.7545935 | -0.8844518 | 6.51E-05 | 0.01164863 | TPPP3 | tubulin polymerization-promoting protein family member 2-like [Xiphophorus hellerii] |
| g1335.t1 | -1.4556611 | 4.55873622 | 3.05E-05 | 0.00689009 | Glrx | glutaredoxin-1 [Xiphophorus hellerii] |
| g13355.t1 | 1.80346958 | 1.27247317 | 0.00033998 | 0.03272745 | Camsap3 | calmodulin-regulated spectrin-associated protein 3-like isoform X1 [Xiphophorus hellerii] |
| g1336.t1 | -1.5717015 | 2.95211954 | 2.91E-05 | 0.0066943 | RHOBTB3 | rho-related BTB domain-containing protein 3 isoform X1 [Xiphophorus maculatus] |
| g13366.t1 | 1.89480049 | 2.38817342 | 3.04E-05 | 0.00689009 | Pdgfa | platelet-derived growth factor subunit A-like [Xiphophorus couchianus] |
| g13484.t1 | 1.73192602 | 3.06169199 | 1.83E-05 | 0.00489846 | SFRP2 | secreted frizzled-related protein 2 [Xiphophorus maculatus] |
| g13603.t1 | -2.1407984 | 2.20563043 | 6.57E-06 | 0.00284794 | ENTPD2 | ectonucleoside triphosphate diphosphohydrolase 2-like [Xiphophorus hellerii] |
| g13611.t1 | 5.76353569 | -2.2153332 | 0.00062864 | 0.04553763 | ADGRD2 | adhesion G-protein coupled receptor D2 [Xiphophorus maculatus] |
| g13632.t1 | 1.75093775 | 4.92674441 | 0.00018833 | 0.02371579 | TNC | tenascin-like isoform X1 [Xiphophorus maculatus] |
| g13659.t1 | 1.67487554 | 3.01916808 | 1.34E-05 | 0.00427327 | SUSD1 | sushi domain-containing protein 1 isoform X1 [Xiphophorus maculatus] |
| g13690.t1 | -2.3515719 | 1.05162574 | 5.46E-05 | 0.01053883 | Abcb9 | ATP-binding cassette sub-family B member 9 [Xiphophorus hellerii] |
| g13701.t1 | -3.1588092 | 0.2087987 | 2.48E-05 | 0.00596371 | Unc5d | netrin receptor UNC5D isoform X1 [Xiphophorus couchianus] |
| g1372.t1 | 1.92691436 | 2.92207372 | 5.30E-05 | 0.01042011 | - | homeobox protein MSX-1 [Xiphophorus maculatus] |
| g13764.t1 | 1.32099442 | 3.87304113 | 0.00012939 | 0.01868259 | DOCK5 | dedicator of cytokinesis protein 5 [Xiphophorus maculatus] |
| g13875.t1 | -3.0920185 | 0.44909622 | 0.00032742 | 0.03194377 | PHYHIP | phytanoyl-CoA hydroxylase-interacting protein-like [Xiphophorus maculatus] |
| g13933.t1 | -2.1958546 | 4.20867465 | 5.05E-09 | 1.21E-05 | - | uncharacterized protein LOC102237790 [Xiphophorus maculatus] |

| | | | | | | |
|---|---|---|---|---|---|---|
| g13975.t1 | 2.04721698 | 3.27696499 | 0.00064413 | 0.04619616 | - | uncharacterized protein LOC102228820 [Xiphophorus maculatus] |
| g13988.t1 | -1.7778122 | 6.4910485 | 0.00018201 | 0.02341734 | unc45b | protein unc-45 homolog B [Xiphophorus couchianus] |
| g14265.t1 | -1.6793706 | 5.64415885 | 0.00011332 | 0.01681062 | Tmed7 | transmembrane emp24 domain-containing protein 7-like [Xiphophorus maculatus] |
| g14283.t1 | 1.45247113 | 2.4615433 | 0.00050544 | 0.04115513 | adra2b | alpha-2B adrenergic receptor-like [Xiphophorus couchianus] |
| g14362.t1 | -3.1222979 | 3.46259099 | 2.28E-05 | 0.0057408 | Psat1 | phosphoserine aminotransferase [Xiphophorus maculatus] |
| g14447.t1 | -1.3577736 | 8.32520611 | 0.00072055 | 0.04896628 | Xbp1 | LOW QUALITY PROTEIN: X-box-binding protein 1 [Xiphophorus maculatus] |
| g14491.t1 | -3.2045365 | 2.39585092 | 1.11E-10 | 4.82E-07 | CCL3 | C-C motif chemokine 3-like [Xiphophorus hellerii] |
| g1450.t1 | 1.9018049 | 2.63554128 | 0.00018272 | 0.02341734 | Fmod | fibromodulin-like [Xiphophorus couchianus] |
| g14563.t1 | -1.5892127 | 5.81375294 | 2.63E-05 | 0.00619889 | PARP14 | protein mono-ADP-ribosyltransferase PARP14-like [Xiphophorus hellerii] |
| g14571.t1 | 1.44293961 | 4.0264958 | 0.00018639 | 0.02360773 | Dmtn | PREDICTED: dematin isoform X1 [Poecilia mexicana] |
| g14605.t1 | -2.4635113 | -0.0054848 | 0.00035038 | 0.03313894 | cfap77 | cilia- and flagella-associated protein 77 [Xiphophorus maculatus] |
| g14610.t1 | -1.5702911 | 6.54247479 | 0.00020038 | 0.02438161 | plpp7 | inactive phospholipid phosphatase 7 [Xiphophorus hellerii] |
| g14689.t1 | -4.2808895 | 2.53130318 | 0.00065758 | 0.04700531 | GDF15 | growth/differentiation factor 15 [Xiphophorus maculatus] |
| g1485.t1 | 3.43362289 | -0.7729182 | 0.00044025 | 0.03798926 | GDF11 | growth/differentiation factor 11-like isoform X1 [Xiphophorus maculatus] |
| g14878.t1 | 3.63482736 | -1.1940578 | 0.00025722 | 0.02827943 | CNMD | leukocyte cell-derived chemotaxin 1 [Xiphophorus maculatus] |
| g149.t1 | 2.47190995 | 3.76512707 | 4.41E-09 | 1.19E-05 | G0s2 | PREDICTED: G0/G1 switch protein 2-like isoform X1 [Poecilia reticulata] |
| g14915.t1 | -2.0448837 | 4.20531053 | 9.35E-06 | 0.00354754 | Dhrs1 | dehydrogenase/reductase SDR family member 1 [Xiphophorus hellerii] |
| g1495.t1 | 6.02181229 | -2.0155611 | 0.00066264 | 0.04705627 | CYP27B1 | 25-hydroxyvitamin D-1 alpha hydroxylase, mitochondrial [Xiphophorus hellerii] |
| g14956.t1 | 2.282118 | 3.14159141 | 0.00014664 | 0.02022922 | xirp1 | xin actin-binding repeat-containing protein 1-like isoform X1 [Xiphophorus maculatus] |
| g14973.t1 | 2.21718408 | 0.56297661 | 8.84E-05 | 0.01386961 | CXCR4 | C-X-C chemokine receptor type 4-like isoform X1 [Xiphophorus couchianus] |
| g14999.t1 | -9.022645 | 0.33549916 | 7.68E-05 | 0.01289941 | Prg4 | proteoglycan 4-like isoform X1 [Xiphophorus hellerii] |
| g150.t1 | 4.91235312 | 0.2993659 | 6.20E-06 | 0.00274116 | CAMK1G | calcium/calmodulin-dependent protein kinase type 1D-like [Xiphophorus couchianus] |
| g15142.t1 | 1.31697025 | 2.79231228 | 0.00057415 | 0.04365748 | tal1 | T-cell acute lymphocytic leukemia protein 1-like isoform X1 [Xiphophorus hellerii] |
| g15163.t1 | 3.99814139 | -1.3962905 | 7.35E-05 | 0.0125431 | AIRE | autoimmune regulator [Xiphophorus maculatus] |
| g15214.t1 | 2.60155274 | 2.0676605 | 1.84E-06 | 0.00117338 | IGFBP3 | insulin-like growth factor-binding protein 3 isoform X1 [Xiphophorus couchianus] |
| g15279.t1 | -1.6051013 | 3.42866253 | 1.01E-05 | 0.00362498 | Higd1a | HIG1 domain family member 1A, mitochondrial [Xiphophorus couchianus] |
| g15360.t1 | 2.03195704 | 2.47505049 | 3.98E-06 | 0.002055 | APOD | apolipoprotein D-like [Xiphophorus maculatus] |
| g15410.t1 | 3.05250978 | 1.1748044 | 1.35E-05 | 0.00427327 | Olfm3 | noelin-3-like [Xiphophorus maculatus] |
| g15497.t1 | -1.3575642 | 4.65497664 | 0.00027771 | 0.02948512 | APOO | MICOS complex subunit MIC26-like [Xiphophorus maculatus] |

| | | | | | | |
|---|---|---|---|---|---|---|
| g15558.t1 | -2.2828918 | 9.4930649 | 0.00037321 | 0.03439698 | cavin4a | PREDICTED: muscle-related coiled-coil protein [Poecilia formosa] |
| g15565.t1 | -1.5245528 | 3.49617779 | 0.00026195 | 0.02836791 | DUSP28 | dual specificity phosphatase 28 [Xiphophorus hellerii] |
| g15655.t1 | -1.8159729 | 2.3369834 | 0.0001972 | 0.02426751 | Zc3hav1 | uncharacterized protein LOC102221414 [Xiphophorus maculatus] |
| g15656.t1 | -2.6477397 | 4.66347072 | 1.05E-09 | 3.25E-06 | Parp12 | zinc finger CCCH-type antiviral protein 1-like [Xiphophorus maculatus] |
| g15689.t1 | 1.53894814 | 2.20211972 | 0.0005378 | 0.04259035 | Cacna1c | voltage-dependent L-type calcium channel subunit alpha-1C-like isoform X7 [Xiphophoru |
| g15764.t1 | 1.23595918 | 3.83758637 | 0.00059603 | 0.04421014 | ZDHHC2 | palmitoyltransferase ZDHHC2 isoform X1 [Xiphophorus hellerii] |
| g1582.t1 | 1.78978153 | 3.74549844 | 5.49E-06 | 0.00253167 | Col7a1 | collagen alpha-1(VII) chain isoform X1 [Xiphophorus hellerii] |
| g16018.t1 | -1.5323631 | 6.56900683 | 0.00029458 | 0.02995459 | Plaat3 | HRAS-like suppressor 3 isoform X1 [Xiphophorus maculatus] |
| g16038.t1 | 1.67119994 | 2.49546032 | 0.00029108 | 0.02995459 | F8 | coagulation factor VIII-like isoform X1 [Xiphophorus hellerii] |
| g16077.t1 | 1.7419836 | 1.6934931 | 0.00026036 | 0.02833755 | SLC43A3 | solute carrier family 43 member 3-like isoform X1 [Xiphophorus maculatus] |
| g16153.t1 | -1.8763922 | 0.76919899 | 0.00072757 | 0.04896628 | Fosl1 | fos-related antigen 1 [Xiphophorus maculatus] |
| g16257.t1 | 1.54941024 | 3.50648414 | 1.74E-05 | 0.00489846 | Rab11fip5 | rab11 family-interacting protein 5 isoform X1 [Xiphophorus maculatus] |
| g16276.t1 | 4.30671165 | -0.9613567 | 0.00030775 | 0.0305763 | Gpc6 | glypican-2 [Xiphophorus couchianus] |
| g16306.t1 | 1.5510953 | 4.11421826 | 1.77E-05 | 0.00489846 | FAT2 | protocadherin Fat 2 [Xiphophorus hellerii] |
| g16332.t1 | -1.557795 | 3.00637173 | 0.00036449 | 0.0341067 | Spata5 | ATPase family protein 2 homolog [Xiphophorus hellerii] |
| g16361.t1 | -1.3822381 | 3.70269879 | 0.00062462 | 0.04553763 | PPID | peptidyl-prolyl cis-trans isomerase D [Xiphophorus couchianus] |
| g16462.t1 | -1.7853076 | 3.58493938 | 0.00012006 | 0.01756997 | Jmjd7 | jmjC domain-containing protein 7 [Xiphophorus maculatus] |
| g16485.t1 | -1.8978059 | 2.27039946 | 7.23E-05 | 0.01242173 | CINP | cyclin-dependent kinase 2-interacting protein isoform X1 [Xiphophorus maculatus] |
| g1649.t1 | -1.5069864 | 3.65897634 | 0.00050355 | 0.04115513 | ndufaf3 | NADH dehydrogenase [ubiquinone] 1 alpha subcomplex assembly factor 3 [Xiphophorus |
| g16536.t1 | 2.27814074 | 7.98738296 | 5.26E-07 | 0.00042193 | CFD | complement factor D-like [Xiphophorus hellerii] |
| g16559.t1 | -1.7415425 | 5.73967709 | 0.00051907 | 0.04179399 | ARG2 | arginase-2, mitochondrial [Xiphophorus couchianus] |
| g16681.t1 | 1.96833642 | 2.91417398 | 1.30E-06 | 0.00087779 | - | A-kinase anchor protein 12-like [Xiphophorus maculatus] |
| g16801.t1 | 1.57153082 | 6.49869331 | 0.00046073 | 0.03852877 | COL10A1 | collagen alpha-1(X) chain-like [Xiphophorus maculatus] |
| g16869.t1 | -2.6667081 | 6.6530047 | 5.97E-06 | 0.00269549 | ODC1 | ornithine decarboxylase [Xiphophorus couchianus] |
| g16954.t1 | -2.7976132 | 1.165352 | 2.14E-05 | 0.00544447 | mfsd2ab | sodium-dependent lysophosphatidylcholine symporter 1-B-like [Xiphophorus maculatus] |
| g17046.t1 | -2.2373465 | 1.40249928 | 3.28E-05 | 0.00723946 | ACYP1 | acylphosphatase-1 [Xiphophorus maculatus] |
| g17081.t1 | -2.779837 | 2.0916888 | 2.12E-05 | 0.00544447 | MSH4 | mutS protein homolog 4 [Xiphophorus maculatus] |
| g17222.t1 | -1.9550472 | 4.60698873 | 1.12E-07 | 0.00016334 | WARS1 | tryptophan--tRNA ligase, cytoplasmic [Xiphophorus couchianus] |
| g17255.t1 | -2.0699986 | 3.70930024 | 7.04E-06 | 0.00288157 | Cmpk2 | UMP-CMP kinase 2, mitochondrial [Xiphophorus maculatus] |

| | | | | | | |
|---|---|---|---|---|---|---|
| g17339.t1 | 6.39538503 | 0.58288704 | 8.13E-05 | 0.01348051 | Hhipl1 | HHIP-like protein 1 [Xiphophorus maculatus] |
| g17447.t1 | -1.7215382 | 4.92134006 | 0.00014841 | 0.02034377 | DPP3 | dipeptidyl peptidase 3 [Xiphophorus maculatus] |
| g17597.t1 | 2.00719967 | 1.00711062 | 0.00025412 | 0.02819525 | Mark4 | PREDICTED: MAP/microtubule affinity-regulating kinase 4-like isoform X5 [Poecilia formo |
| g17682.t1 | -7.0231593 | -1.5247546 | 0.00071768 | 0.04896628 | HSF5 | heat shock factor protein 5 [Xiphophorus hellerii] |
| g17733.t1 | -1.7590158 | 4.64769702 | 3.19E-06 | 0.00177408 | banf1 | barrier-to-autointegration factor [Xiphophorus maculatus] |
| g17755.t1 | 6.55794203 | -1.6393712 | 0.00014969 | 0.02039041 | Kcnip1 | Kv channel-interacting protein 1-like isoform X1 [Xiphophorus maculatus] |
| g17795.t1 | -2.157838 | 5.22593205 | 6.62E-05 | 0.01171157 | RTN2 | reticulon-2-like [Xiphophorus maculatus] |
| g1790.t1 | 2.50626535 | 0.82613938 | 0.00016252 | 0.02146416 | A3galt2 | alpha-1,3-galactosyltransferase 2-like isoform X1 [Xiphophorus hellerii] |
| g1795.t1 | -1.9418687 | 1.81257884 | 0.00039159 | 0.03533917 | MIIP | migration and invasion-inhibitory protein isoform X1 [Xiphophorus couchianus] |
| g17951.t1 | 2.04613731 | 0.51928277 | 0.00057648 | 0.04365748 | Nrxn2 | neurexin-2-like isoform X3 [Xiphophorus maculatus] |
| g17997.t1 | -1.4323123 | 3.03380986 | 0.00041205 | 0.03662146 | HEPHL1 | hephaestin-like protein 1 [Xiphophorus maculatus] |
| g18045.t1 | -1.9842005 | 4.05931537 | 7.11E-06 | 0.00288157 | secA | uncharacterized protein LOC116712644 [Xiphophorus hellerii] |
| g1812.t1 | 1.76160673 | 3.84620635 | 0.00017887 | 0.02324205 | IFFO2 | intermediate filament family orphan 2-like isoform X1 [Xiphophorus couchianus] |
| g18127.t1 | 3.87998418 | -0.3351838 | 9.17E-05 | 0.01428109 | Gria4 | glutamate receptor 4 isoform X1 [Xiphophorus maculatus] |
| g18162.t1 | 1.73922969 | 3.58328611 | 8.61E-05 | 0.01382105 | Clip2 | CAP-Gly domain-containing linker protein 2 isoform X2 [Xiphophorus hellerii] |
| g18168.t1 | -2.6008674 | 1.18234467 | 1.02E-05 | 0.00362498 | - | - |
| g18447.t1 | 3.08672178 | 0.18226785 | 4.70E-05 | 0.00969078 | PLEKHF1 | pleckstrin homology domain-containing family F member 2-like [Xiphophorus maculatus] |
| g1855.t1 | -1.7442586 | 4.18140114 | 2.31E-05 | 0.00574308 | mars1 | methionine--tRNA ligase, cytoplasmic isoform X1 [Xiphophorus maculatus] |
| g18574.t1 | -1.7340501 | 2.04025576 | 0.00034369 | 0.03279266 | RBBP6 | E3 ubiquitin-protein ligase RBBP6 isoform X1 [Xiphophorus maculatus] |
| g18630.t1 | -3.4604879 | -0.0846888 | 9.51E-06 | 0.00354754 | MPRIP | myosin-14-like isoform X2 [Xiphophorus hellerii] |
| g1868.t1 | -1.7986927 | 3.87562823 | 0.00014248 | 0.01978207 | PRELID3B | PRELI domain containing protein 3B-like [Xiphophorus couchianus] |
| g18721.t1 | -2.2733693 | 14.5154393 | 0.00023816 | 0.02788217 | prvb | parvalbumin beta 2-like [Xiphophorus maculatus] |
| g18817.t1 | -2.2708535 | 0.85312945 | 0.00036794 | 0.034203 | Rasd1 | dexamethasone-induced Ras-related protein 1-like [Xiphophorus couchianus] |
| g18821.t1 | 1.26570308 | 4.20500467 | 0.00063196 | 0.04562574 | sox9-b | transcription factor Sox-9-like [Xiphophorus couchianus] |
| g18844.t1 | -1.9160315 | 6.24487822 | 0.0002197 | 0.02643563 | SOCS3 | suppressor of cytokine signaling 3-like [Xiphophorus maculatus] |
| g18862.t1 | -2.3899305 | 1.56512368 | 0.00037964 | 0.03443953 | PDE6H | retinal cone rhodopsin-sensitive cGMP 3',5'-cyclic phosphodiesterase subunit gamma-lik |
| g18872.t1 | 2.20599271 | 1.19063169 | 0.00044769 | 0.03842684 | HS3ST3B1 | heparan sulfate glucosamine 3-O-sulfotransferase 3B1-like [Xiphophorus couchianus] |
| g1888.t1 | 1.57685406 | 3.39778758 | 0.00018484 | 0.02355016 | TFAP2C | transcription factor AP-2-alpha-like isoform X2 [Xiphophorus maculatus] |
| g18935.t1 | 1.80740946 | 7.20062411 | 0.00010315 | 0.01562275 | HEBP2 | heme-binding protein 2-like [Xiphophorus maculatus] |

| | | | | | | |
|---|---|---|---|---|---|---|
| g18973.t1 | 1.80739016 | 4.18032243 | 9.37E-07 | 0.00065469 | hbb1 | hemoglobin subunit beta-1-like [Xiphophorus couchianus] |
| g19051.t1 | 1.6431078 | 3.35888433 | 0.00033079 | 0.03212803 | C1QL2 | complement C1q-like protein 2 [Xiphophorus maculatus] |
| g191.t1 | 4.97824285 | -0.4983862 | 8.92E-06 | 0.00351312 | - | uncharacterized protein LOC111608151 [Xiphophorus maculatus] |
| g19261.t1 | 2.70107898 | 5.67467941 | 1.84E-10 | 6.64E-07 | suz12 | polycomb protein suz12-B-like [Xiphophorus maculatus] |
| g1933.t1 | -1.987907 | 3.36604927 | 4.94E-07 | 0.00041143 | RBM12 | RNA-binding protein 12 [Xiphophorus hellerii] |
| g19387.t1 | -1.8586288 | 2.33810843 | 8.36E-05 | 0.01361377 | MMP24 | matrix metalloproteinase-24 [Xiphophorus maculatus] |
| g19420.t1 | 1.21471128 | 4.09242239 | 0.00055712 | 0.0432495 | ripor3 | RIPOR family member 3 isoform X2 [Xiphophorus couchianus] |
| g19441.t1 | -4.5233201 | 4.86763125 | 5.07E-08 | 8.44E-05 | LOC4327591 | ADP-ribosylation factor 1-like [Xiphophorus maculatus] |
| g19479.t1 | 4.32472186 | -1.653311 | 0.00071721 | 0.04896628 | SYT1 | synaptotagmin-1 isoform X2 [Xiphophorus maculatus] |
| g19501.t1 | 1.68376823 | 2.34933589 | 4.18E-05 | 0.00899031 | MAGI2 | membrane-associated guanylate kinase, WW and PDZ domain-containing protein 2 isofo |
| g19534.t1 | 1.34856664 | 3.33661788 | 0.00042318 | 0.03741065 | SLITRK6 | SLIT and NTRK-like protein 6 [Xiphophorus hellerii] |
| g19577.t1 | 1.9708837 | 1.30623336 | 0.00040394 | 0.03615278 | Gpr137b | integral membrane protein GPR137B-like [Xiphophorus hellerii] |
| g1973.t1 | 1.84006182 | 3.14487737 | 2.33E-05 | 0.00574308 | Kif17 | kinesin-like protein KIF17 isoform X1 [Xiphophorus maculatus] |
| g19739.t1 | -1.4267719 | 3.25515886 | 0.00037895 | 0.03443953 | Znrd1 | DNA-directed RNA polymerase I subunit RPA12 [Xiphophorus couchianus] |
| g19834.t1 | 1.52845928 | 6.17654171 | 0.00022215 | 0.02658323 | - | glutathione S-transferase A-like [Xiphophorus maculatus] |
| g19892.t1 | 2.04230322 | 2.84089916 | 4.11E-07 | 0.00037149 | QNR-71 | transmembrane glycoprotein NMB isoform X1 [Xiphophorus couchianus] |
| g19911.t1 | -1.7276334 | 4.48716449 | 8.68E-05 | 0.01382297 | BET1 | BET1 homolog [Xiphophorus maculatus] |
| g19925.t1 | -2.3786567 | 1.79795764 | 6.06E-05 | 0.01112812 | btd | biotinidase [Xiphophorus hellerii] |
| g20044.t1 | -6.8433863 | -1.6602968 | 5.25E-05 | 0.01042011 | Stmnd1 | stathmin domain-containing protein 1 [Xiphophorus maculatus] |
| g2025.t1 | -5.2241321 | -0.9546425 | 0.00055331 | 0.04310838 | SYCP1 | synaptonemal complex protein 1 isoform X2 [Xiphophorus couchianus] |
| g20277.t1 | -1.8422099 | 5.50387588 | 1.48E-05 | 0.00446455 | mllt11 | protein AF1q [Xiphophorus maculatus] |
| g20300.t1 | -1.4688049 | 4.69671582 | 0.00074286 | 0.04965912 | - | uncharacterized protein LOC114155365 [Xiphophorus couchianus] |
| g20315.t1 | 1.39945012 | 3.67303764 | 0.00030644 | 0.0305763 | COL14A1 | collagen alpha-1(XIV) chain-like isoform X1 [Xiphophorus couchianus] |
| g20320.t1 | 1.64145218 | 4.80840518 | 0.00029189 | 0.02995459 | Enpp2 | ectonucleotide pyrophosphatase/phosphodiesterase family member 2-like [Xiphophorus |
| g2034.t1 | -6.1107231 | -2.2409355 | 0.00058926 | 0.04405845 | ADORA1 | adenosine receptor A1-like [Xiphophorus couchianus] |
| g20348.t1 | -7.9592993 | -0.7164144 | 1.53E-05 | 0.00452321 | Cox6b1 | cytochrome c oxidase subunit 6B1-like [Xiphophorus couchianus] |
| g20418.t1 | -1.1839077 | 4.67112077 | 0.00075217 | 0.04997306 | pithd1 | PITH domain-containing protein 1 [Xiphophorus maculatus] |
| g20445.t1 | -6.3200541 | 1.04180299 | 0.00028932 | 0.02995459 | apoeb | apolipoprotein Eb-like [Xiphophorus couchianus] |
| g20447.t1 | -7.9621095 | -0.6723121 | 0.00024282 | 0.02806153 | - | apolipoprotein A-IV-like [Xiphophorus couchianus] |

| | | | | | | |
|---|---|---|---|---|---|---|
| g2050.t1 | -4.5052256 | -1.4614167 | 4.84E-05 | 0.00979177 | - | elastase-1-like [Xiphophorus maculatus] |
| g20584.t1 | -2.4934485 | 3.86076504 | 5.83E-05 | 0.01089624 | tmem41ab | transmembrane protein 41A-A-like [Xiphophorus hellerii] |
| g20627.t1 | -6.9378358 | -1.5968941 | 0.00034192 | 0.03276816 | eno4 | enolase 4 [Xiphophorus maculatus] |
| g20638.t1 | 1.55525878 | 2.17469388 | 0.00024631 | 0.02806153 | SLC2A9 | solute carrier family 2, facilitated glucose transporter member 9-like [Xiphophorus couch |
| g20660.t1 | -3.0328109 | 4.51083423 | 0.00023763 | 0.02788217 | Peli1 | E3 ubiquitin-protein ligase pellino homolog 1 [Xiphophorus maculatus] |
| g20687.t1 | -3.2133786 | 0.4523132 | 0.00055115 | 0.0430951 | MAL | myelin and lymphocyte protein-like isoform X2 [Xiphophorus hellerii] |
| g20707.t1 | 1.69390098 | 2.99864504 | 2.46E-05 | 0.00596371 | Ccdc88a | girdin isoform X1 [Xiphophorus maculatus] |
| g20842.t1 | 2.25450574 | 1.8824651 | 1.39E-05 | 0.00427327 | AKR1B1 | aldose reductase-like [Xiphophorus maculatus] |
| g21094.t1 | -8.6115639 | 0.02745668 | 8.30E-07 | 0.00061962 | - | chymotrypsin A-like [Xiphophorus couchianus] |
| g21120.t1 | 1.27879552 | 4.40677103 | 0.00067459 | 0.04728494 | gata3 | trans-acting T-cell-specific transcription factor GATA-3 isoform X1 [Xiphophorus maculatu |
| g21130.t1 | 1.9654861 | 2.52443569 | 8.19E-05 | 0.01348051 | D2 | cAMP-regulated D2 protein-like [Xiphophorus maculatus] |
| g21178.t1 | 1.74563737 | 3.60450449 | 4.23E-05 | 0.00899031 | FHL2 | four and a half LIM domains protein 2-like [Xiphophorus hellerii] |
| g21189.t1 | 1.46219714 | 3.45770654 | 5.25E-05 | 0.01042011 | - | growth hormone receptor-like isoform X1 [Xiphophorus maculatus] |
| g21222.t1 | -1.3339608 | 3.97158968 | 0.00043477 | 0.03772453 | - | uncharacterized protein LOC114140628 isoform X2 [Xiphophorus couchianus] |
| g228.t1 | 1.60522735 | 2.13145039 | 0.00025152 | 0.02819525 | Mmp19 | matrix metalloproteinase-19-like [Xiphophorus maculatus] |
| g2303.t1 | 2.17026186 | 1.07104249 | 0.00045242 | 0.03842684 | PTPN13 | tyrosine-protein phosphatase non-receptor type 13-like isoform X2 [Xiphophorus couchi |
| g2352.t1 | -5.4572601 | 0.92157941 | 1.78E-13 | 3.86E-09 | Eloa | elongin-A-like [Xiphophorus maculatus] |
| g2456.t1 | 1.6036293 | 3.28186809 | 8.61E-05 | 0.01382105 | Vit | vitrin isoform X3 [Xiphophorus hellerii] |
| g2462.t1 | 9.18549368 | 0.610177 | 0.00041256 | 0.03662146 | Eif2ak2 | PREDICTED: interferon-induced, double-stranded RNA-activated protein kinase isoform X |
| g2720.t1 | -1.3013859 | 4.20970819 | 0.00046056 | 0.03852877 | dohh | deoxyhypusine hydroxylase [Xiphophorus maculatus] |
| g2775.t1 | 1.89537859 | 2.60990326 | 0.00043121 | 0.03772453 | sox14 | transcription factor Sox-14 [Xiphophorus maculatus] |
| g2906.t1 | -1.849187 | 2.42007792 | 0.00020023 | 0.02438161 | REM2 | GTP-binding protein REM 2-like [Xiphophorus maculatus] |
| g2941.t1 | 1.51639876 | 5.22698632 | 4.22E-05 | 0.00899031 | SUCO | SUN domain-containing ossification factor isoform X1 [Xiphophorus maculatus] |
| g3058.t1 | -2.400748 | 3.0411481 | 7.18E-06 | 0.00288157 | AMH | PREDICTED: muellerian-inhibiting factor [Poecilia reticulata] |
| g3071.t1 | -1.5131068 | 4.60979242 | 0.00050913 | 0.04130066 | sec22bb | vesicle-trafficking protein SEC22b-B isoform X2 [Xiphophorus maculatus] |
| g309.t1 | -3.2997109 | 0.44035181 | 0.00072183 | 0.04896628 | Tmpo | lamina-associated polypeptide 2, isoforms beta/gamma-like isoform X1 [Xiphophorus he |
| g3092.t1 | -2.7201969 | 3.85905988 | 4.91E-06 | 0.00236252 | ERICH3 | glutamate-rich protein 3 [Xiphophorus maculatus] |
| g32.t1 | -1.429168 | 6.3183432 | 0.00058991 | 0.04405845 | SRPK3 | SRSF protein kinase 3-like isoform X3 [Xiphophorus hellerii] |
| g321.t1 | -1.4138453 | 7.52225294 | 0.00069086 | 0.04795909 | lmod3 | leiomodin-3 [Xiphophorus maculatus] |

| | | | | | | |
|---|---|---|---|---|---|---|
| g3224.t1 | 3.49191062 | 1.94759189 | 6.23E-05 | 0.01133052 | Obscn | obscurin-like isoform X1 [Xiphophorus couchianus] |
| g3374.t1 | -1.4704953 | 5.09155569 | 0.00011206 | 0.01673858 | MYLK4 | myosin light chain kinase family member 4 isoform X1 [Xiphophorus couchianus] |
| g3406.t1 | -1.884424 | 6.94056347 | 1.40E-05 | 0.00427327 | gamt | guanidinoacetate N-methyltransferase [Xiphophorus couchianus] |
| g3547.t1 | -1.6866491 | 2.80737357 | 0.00045002 | 0.03842684 | tmem69 | transmembrane protein 69-like [Xiphophorus couchianus] |
| g3613.t1 | 1.67735335 | 5.52727301 | 1.72E-05 | 0.00489846 | KIFAP3 | kinesin-associated protein 3 isoform X1 [Xiphophorus maculatus] |
| g3637.t1 | -1.5313073 | 5.22771389 | 0.00066001 | 0.04702339 | NRDC | nardilysin-like isoform X1 [Xiphophorus hellerii] |
| g3699.t1 | 2.61014409 | 3.05811711 | 0.00014248 | 0.01978207 | CNDP2 | cytosolic non-specific dipeptidase-like [Xiphophorus maculatus] |
| g3969.t1 | -5.8533317 | 1.90191444 | 5.47E-06 | 0.00253167 | Try3 | trypsin-3-like [Xiphophorus couchianus] |
| g4020.t1 | 3.05649739 | 1.80945939 | 1.13E-07 | 0.00016334 | TNC | tenascin-like [Xiphophorus couchianus] |
| g406.t1 | 2.17890527 | 2.80378198 | 2.72E-05 | 0.00632892 | Wnt7a | protein Wnt-7a [Xiphophorus maculatus] |
| g4269.t1 | 1.82507922 | 7.89504767 | 2.00E-05 | 0.0052547 | Apoh | beta-2-glycoprotein 1-like isoform X2 [Xiphophorus maculatus] |
| g4273.t1 | 2.38558117 | 1.73187663 | 0.00026722 | 0.02879473 | GRIN2D | glutamate receptor ionotropic, NMDA 2D-like isoform X1 [Xiphophorus maculatus] |
| g4274.t1 | 2.54485459 | 1.18225148 | 0.00053472 | 0.04257914 | PRKCG | protein kinase C gamma type-like isoform X1 [Xiphophorus couchianus] |
| g4364.t1 | -1.583603 | 6.3971633 | 0.00030604 | 0.0305763 | vars1 | valine--tRNA ligase [Xiphophorus maculatus] |
| g4399.t1 | 1.39688874 | 5.78474747 | 0.00025879 | 0.02830826 | LIPE | hormone-sensitive lipase isoform X1 [Xiphophorus couchianus] |
| g4405.t1 | 1.67719903 | 3.36596207 | 1.83E-05 | 0.00489846 | MEP1B | meprin A subunit beta-like [Xiphophorus maculatus] |
| g4432.t1 | -1.5625273 | 3.89095678 | 4.82E-05 | 0.00979177 | DHDDS | dehydrodolichyl diphosphate synthase complex subunit DHDDS [Xiphophorus hellerii] |
| g4485.t1 | 2.89577546 | -0.7108637 | 0.00060263 | 0.04454737 | Foxo6 | forkhead box protein O3-like [Xiphophorus hellerii] |
| g4498.t1 | 1.55265629 | 6.30473149 | 0.00059318 | 0.04414991 | - | apolipoprotein C-I [Xiphophorus hellerii] |
| g4735.t1 | -2.4564758 | 0.65755791 | 5.34E-05 | 0.01042011 | IGDCC3 | immunoglobulin superfamily DCC subclass member 3 [Xiphophorus maculatus] |
| g4752.t1 | -3.3845648 | 0.99006549 | 0.00062359 | 0.04553763 | - | L-amino-acid oxidase-like isoform X1 [Xiphophorus couchianus] |
| g4759.t1 | 1.37230661 | 3.05548242 | 0.00054752 | 0.0430951 | - | colorectal cancer-associated protein 2 isoform X1 [Xiphophorus maculatus] |
| g4765.t1 | 2.44006495 | 4.0110796 | 2.25E-11 | 1.22E-07 | BCO2 | beta,beta-carotene 9',10'-oxygenase-like isoform X1 [Xiphophorus couchianus] |
| g4792.t1 | 1.458005 | 2.91955549 | 0.00053879 | 0.04259035 | Fat3 | protocadherin Fat 3 isoform X1 [Xiphophorus couchianus] |
| g4847.t1 | 1.52400791 | 3.7798773 | 0.00039666 | 0.03564876 | DHRS11 | dehydrogenase/reductase SDR family member 11-like [Xiphophorus hellerii] |
| g4948.t1 | -1.6116926 | 3.12886485 | 0.00072818 | 0.04896628 | ARL14 | ADP-ribosylation factor-like protein 14 [Xiphophorus couchianus] |
| g5002.t1 | -2.2073284 | 4.94672308 | 0.00027361 | 0.02919291 | Rtn2 | reticulon-2-like isoform X1 [Xiphophorus couchianus] |
| g5078.t1 | 1.43553074 | 3.58832425 | 0.00015118 | 0.02046439 | VTN | vitronectin isoform X1 [Xiphophorus hellerii] |
| g5092.t1 | 2.79208669 | 1.0548886 | 3.86E-07 | 0.00037149 | SLCO1C1 | solute carrier organic anion transporter family member 1C1-like [Xiphophorus hellerii] |

| | | | | | | |
|---|---|---|---|---|---|---|
| g5205.t1 | -3.4577183 | -1.1791841 | 0.00024501 | 0.02806153 | - | immunoglobulin superfamily member 5-like isoform X1 [Xiphophorus couchianus] |
| g5294.t1 | 3.11390236 | 5.51688075 | 2.07E-06 | 0.00127964 | apoa1 | apolipoprotein A-I [Xiphophorus maculatus] |
| g5333.t1 | -1.9064673 | 4.08471263 | 3.15E-07 | 0.00032452 | Tapbpl | tapasin-like [Xiphophorus maculatus] |
| g534.t1 | 2.44793265 | -0.4035694 | 0.00045449 | 0.03842684 | ATP6AP1 | V-type proton ATPase subunit S1-like [Xiphophorus maculatus] |
| g5442.t1 | -4.0217832 | 0.80426737 | 0.00028762 | 0.02995459 | - | tubulin beta-4B chain isoform X7 [Echeneis naucrates] |
| g5666.t1 | 2.62510691 | 2.90580254 | 0.00015341 | 0.0206375 | Tmc3 | transmembrane channel-like protein 3 isoform X3 [Xiphophorus maculatus] |
| g5672.t1 | 2.34606468 | 1.5806621 | 0.00014245 | 0.01978207 | Cemip | cell migration-inducing and hyaluronan-binding protein isoform X1 [Xiphophorus hellerii] |
| g5791.t1 | -2.8492977 | 2.75470809 | 1.85E-07 | 0.00023621 | IDH2 | isocitrate dehydrogenase [NADP], mitochondrial-like [Xiphophorus maculatus] |
| g5903.t1 | 6.65289906 | -1.6196037 | 0.00032642 | 0.03194377 | NHS | Nance-Horan syndrome protein-like isoform X1 [Xiphophorus maculatus] |
| g6009.t1 | -1.2936087 | 4.25153298 | 0.00050171 | 0.04115513 | MPHOSPH10 | U3 small nucleolar ribonucleoprotein protein MPP10 [Xiphophorus couchianus] |
| g6122.t1 | 1.32991609 | 4.54674022 | 0.00062759 | 0.04553763 | Tex9 | testis-expressed protein 9-like [Xiphophorus hellerii] |
| g6153.t1 | 2.23603624 | 2.06919689 | 0.00045505 | 0.03842684 | Strc | stereocilin [Xiphophorus couchianus] |
| g6186.t1 | 1.31352585 | 7.11453092 | 0.00062165 | 0.04553763 | Ppfibp2 | liprin-beta-2 isoform X1 [Xiphophorus hellerii] |
| g6256.t1 | 2.84643204 | 1.32172534 | 2.18E-06 | 0.00131136 | Kif26b | kinesin-like protein KIF26B isoform X1 [Xiphophorus maculatus] |
| g6321.t1 | 2.83306216 | 1.5202185 | 2.93E-06 | 0.00167596 | SYT11 | synaptotagmin-4-like [Xiphophorus hellerii] |
| g6493.t1 | -2.0365944 | 6.20737993 | 3.98E-06 | 0.002055 | Kiaa0895l | uncharacterized protein KIAA0895-like homolog [Xiphophorus hellerii] |
| g6497.t1 | -1.346115 | 3.92367653 | 0.00023953 | 0.02789205 | Rora | nuclear receptor ROR-alpha A-like isoform X1 [Xiphophorus couchianus] |
| g6561.t1 | -2.2663946 | 3.70725636 | 1.06E-05 | 0.00366959 | noct | nocturnin isoform X1 [Xiphophorus maculatus] |
| g6573.t1 | -1.6337066 | 3.7598446 | 4.44E-06 | 0.00218554 | zgc:153146 | UPF0602 protein C4orf47 homolog [Xiphophorus hellerii] |
| g6659.t1 | -1.2730353 | 3.78664193 | 0.00075054 | 0.04997306 | wdr4 | tRNA (guanine-N(7)-)-methyltransferase non-catalytic subunit WDR4 isoform X1 [Xiphop |
| g6741.t1 | -3.4107433 | 2.05299654 | 4.95E-12 | 5.36E-08 | - | uncharacterized protein LOC114147991 isoform X1 [Xiphophorus couchianus] |
| g6742.t1 | -1.873864 | 1.76336378 | 0.00021551 | 0.02607662 | - | PREDICTED: uncharacterized protein LOC106911626 [Poecilia mexicana] |
| g6772.t1 | -1.2652507 | 4.27792047 | 0.00051247 | 0.04141661 | popdc2 | popeye domain-containing protein 2 isoform X1 [Xiphophorus couchianus] |
| g6778.t1 | -1.752914 | 3.77057055 | 9.52E-06 | 0.00354754 | IL1R1 | interleukin-1 receptor type 1-like isoform X1 [Xiphophorus couchianus] |
| g6979.t1 | 3.12056596 | 1.01140547 | 4.12E-07 | 0.00037149 | CBS | cystathionine beta-synthase-like [Xiphophorus hellerii] |
| g6988.t1 | -1.6523055 | 5.27617933 | 0.00024441 | 0.02806153 | Irs2 | insulin receptor substrate 2-like isoform X1 [Xiphophorus couchianus] |
| g7016.t1 | -2.1682297 | 4.09857216 | 6.02E-07 | 0.00046581 | ACP6 | lysophosphatidic acid phosphatase type 6 [Xiphophorus couchianus] |
| g7145.t1 | 3.18073458 | -0.0186609 | 0.00011639 | 0.01714962 | CERS6 | ceramide synthase 6 [Xiphophorus couchianus] |
| g7178.t1 | 3.61813499 | 0.91730018 | 0.00072028 | 0.04896628 | ACVR1C | activin receptor type-1C [Xiphophorus maculatus] |

| | | | | | | |
|---|---|---|---|---|---|---|
| g7211.t1 | 3.61146121 | -0.0424762 | 3.26E-05 | 0.00723946 | trpm2 | transient receptor potential cation channel subfamily M member 2-like isoform X1 [Xipho |
| g7229.t1 | 1.50669982 | 2.84796027 | 0.00057169 | 0.04365748 | PTPRN | receptor-type tyrosine-protein phosphatase-like N isoform X1 [Xiphophorus maculatus] |
| g7310.t1 | 2.31663257 | 2.18968967 | 1.38E-05 | 0.00427327 | wnt10a | protein Wnt-10a [Xiphophorus couchianus] |
| g7363.t1 | 7.88483422 | -0.5922262 | 4.69E-08 | 8.44E-05 | Hspa12a | heat shock 70 kDa protein 12A-like [Xiphophorus maculatus] |
| g7365.t1 | 6.37552808 | -1.8001834 | 6.42E-05 | 0.01159336 | Hspa12a | heat shock 70 kDa protein 12A-like [Xiphophorus maculatus] |
| g7367.t1 | 7.96356395 | -0.5412933 | 1.57E-07 | 0.0002127 | Hspa12a | heat shock 70 kDa protein 12A-like isoform X1 [Xiphophorus couchianus] |
| g7369.t1 | 7.67786162 | -0.769547 | 4.10E-08 | 8.07E-05 | Hspa12a | heat shock 70 kDa protein 12A-like isoform X3 [Xiphophorus couchianus] |
| g7370.t1 | 7.43204959 | -0.9749777 | 2.83E-07 | 0.00030594 | HSPA12A | heat shock 70 kDa protein 12A-like isoform X5 [Xiphophorus couchianus] |
| g743.t1 | 2.30251546 | 0.67293378 | 0.00053046 | 0.04244673 | Epb41l1 | band 4.1-like protein 1 isoform X1 [Xiphophorus couchianus] |
| g7441.t1 | 1.89331759 | 4.68304173 | 0.00015788 | 0.02097925 | gdf-8 | growth/differentiation factor 8 [Xiphophorus hellerii] |
| g7542.t1 | 1.41388115 | 2.70733244 | 0.000575 | 0.04365748 | ALOX5 | PREDICTED: arachidonate 12-lipoxygenase, 12S-type-like isoform X1 [Poecilia reticulata] |
| g7552.t1 | 2.99025531 | -0.3813993 | 5.65E-05 | 0.01072569 | - | uncharacterized protein LOC114158063 [Xiphophorus couchianus] |
| g7625.t1 | 1.96194711 | 1.43016929 | 0.00017921 | 0.02324205 | znf219 | zinc finger protein 219-like [Xiphophorus couchianus] |
| g7768.t1 | -1.5728712 | 7.33314903 | 0.00022541 | 0.02682522 | txn | thioredoxin-like [Xiphophorus couchianus] |
| g7926.t1 | 2.05688135 | 2.81131289 | 2.58E-07 | 0.00029395 | PPARGC1A | peroxisome proliferator-activated receptor gamma coactivator 1-alpha isoform X2 [Xipho |
| g7941.t1 | -4.0187115 | 0.61889678 | 0.00057412 | 0.04365748 | - | uncharacterized protein LOC116733029 isoform X1 [Xiphophorus hellerii] |
| g7995.t1 | -3.2432415 | -0.9545066 | 5.98E-05 | 0.01107815 | - | basic leucine zipper transcriptional factor ATF-like 2 [Xiphophorus hellerii] |
| g8159.t1 | -3.7366085 | 2.23634982 | 1.01E-11 | 7.27E-08 | - | uncharacterized protein LOC116732639 [Xiphophorus hellerii] |
| g8232.t1 | 1.4664076 | 2.61915121 | 0.00038001 | 0.03443953 | HBAD | hemoglobin subunit alpha-D-like isoform X2 [Xiphophorus hellerii] |
| g8339.t1 | -1.9063881 | 5.53434441 | 3.74E-05 | 0.00819072 | - | stonustoxin subunit beta-like [Xiphophorus hellerii] |
| g8400.t1 | -1.4928187 | 4.75871309 | 9.39E-05 | 0.01447799 | pelo | PREDICTED: protein pelota homolog [Poecilia mexicana] |
| g8519.t1 | 6.28611545 | -1.8222635 | 0.00033996 | 0.03272745 | PPA1 | inorganic pyrophosphatase-like [Xiphophorus hellerii] |
| g8606.t1 | -2.1778943 | 3.71119651 | 2.94E-06 | 0.00167596 | chac2 | glutathione-specific gamma-glutamylcyclotransferase 2 [Xiphophorus couchianus] |
| g8607.t1 | 1.95218136 | 1.780967 | 6.82E-05 | 0.01190836 | psme4b | proteasome activator complex subunit 4B-like [Xiphophorus maculatus] |
| g8616.t1 | 1.54734364 | 3.89682671 | 1.80E-05 | 0.00489846 | - | uncharacterized protein LOC114152212 isoform X1 [Xiphophorus couchianus] |
| g8661.t1 | -1.9137223 | 3.19690813 | 0.00068085 | 0.04750372 | Map3k14 | mitogen-activated protein kinase kinase kinase 14 [Xiphophorus maculatus] |
| g8702.t1 | -3.5099077 | 2.91482521 | 8.11E-09 | 1.76E-05 | g6pc3 | glucose-6-phosphatase 3 [Xiphophorus maculatus] |
| g9122.t1 | 2.4357664 | 0.78522986 | 9.43E-05 | 0.01447799 | BICD1 | protein bicaudal D homolog 1-like isoform X1 [Xiphophorus maculatus] |
| g9124.t1 | 5.35415837 | -0.978573 | 1.13E-05 | 0.00383393 | SLC23A2 | solute carrier family 23 member 1-like [Xiphophorus hellerii] |

| | | | | | | |
|---|---|---|---|---|---|---|
| g9138.t1 | -1.5976727 | 5.47557075 | 9.98E-05 | 0.01522117 | NUTF2 | nuclear transport factor 2 [Xiphophorus maculatus] |
| g9159.t1 | -1.4259828 | 3.71104787 | 0.00025322 | 0.02819525 | setd6 | N-lysine methyltransferase SETD6 isoform X2 [Xiphophorus hellerii] |
| g9162.t1 | 1.17818248 | 3.45489904 | 0.00070172 | 0.04855771 | znf423 | zinc finger protein 423 isoform X1 [Xiphophorus hellerii] |
| g9185.t1 | 2.22598261 | 2.07637276 | 3.78E-06 | 0.00204884 | IL34 | interleukin-34 [Xiphophorus maculatus] |
| g9285.t1 | 1.39919067 | 4.16459224 | 0.00047154 | 0.03928128 | MYO5A | unconventional myosin-Va-like isoform X1 [Xiphophorus couchianus] |
| g9385.t1 | -2.2922123 | 3.88374354 | 9.26E-07 | 0.00065469 | FBXL22 | LOW QUALITY PROTEIN: F-box and leucine-rich protein 22 [Xiphophorus hellerii] |
| g9426.t1 | -2.2299222 | 2.55567064 | 0.00036533 | 0.0341067 | FRMD5 | FERM domain-containing protein 5-like isoform X1 [Xiphophorus couchianus] |
| g9430.t1 | -4.2759161 | -1.7790189 | 0.0006821 | 0.04750372 | ankrd34b | ankyrin repeat domain-containing protein 34C [Xiphophorus maculatus] |
| g9820.t1 | 1.53774857 | 4.01849842 | 0.00024746 | 0.02806153 | cntn1a | contactin-1a-like isoform X1 [Xiphophorus hellerii] |
| g9877.t1 | -1.3198341 | 5.19406176 | 0.00058268 | 0.04382031 | AEN | apoptosis-enhancing nuclease [Xiphophorus hellerii] |
| g9957.t1 | 1.5017585 | 4.00299383 | 7.49E-05 | 0.01267398 | Cspg4 | chondroitin sulfate proteoglycan 4 [Xiphophorus hellerii] |

**Figure S2** Activity level test tank

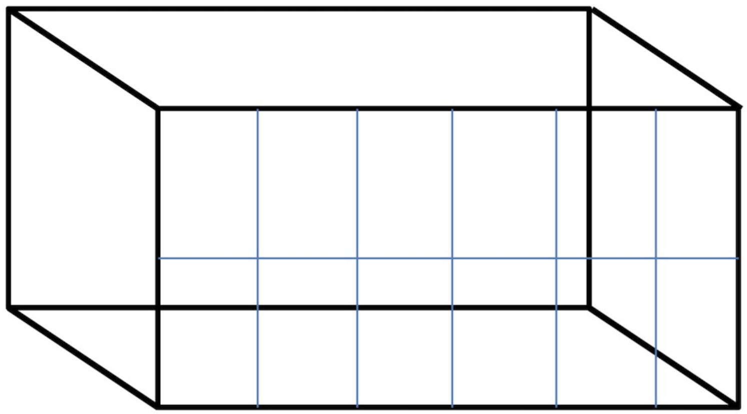

**Figure S3** The relationship between relative nuchal hump size and activity levels by treatment; blue = cold, red = warm

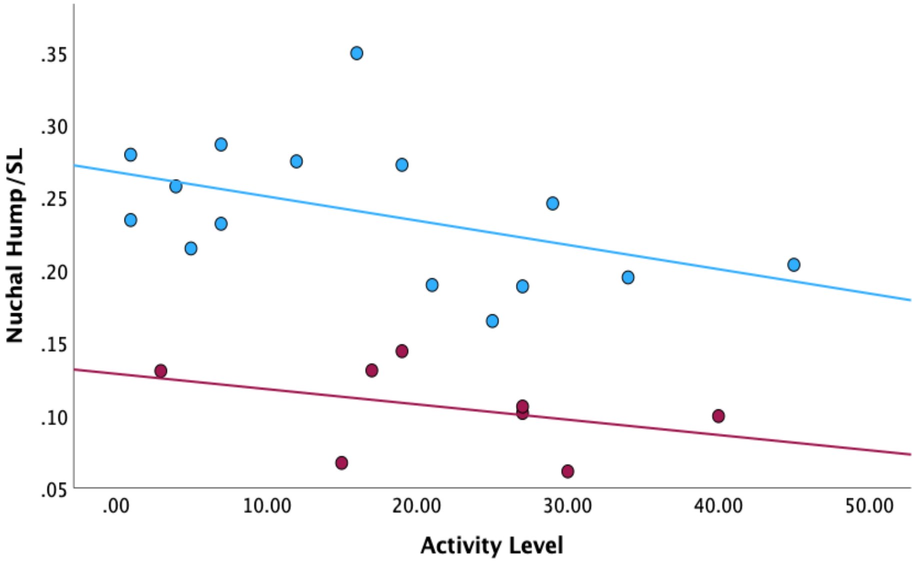

Supplement: Supplementary file 1 — Supplementary file1 (PDF 765 KB) [file 10695_2025_1539_MOESM1_ESM.pdf]
